# Red Blood Cell Classification Based on Attention Residual Feature Pyramid Network

**DOI:** 10.3389/fmed.2021.741407

**Published:** 2021-12-14

**Authors:** Weiqing Song, Pu Huang, Jing Wang, Yajuan Shen, Jian Zhang, Zhiming Lu, Dengwang Li, Danhua Liu

**Affiliations:** ^1^Shandong Key Laboratory of Medical Physics and Image Processing, Shandong Institute of Industrial Technology for Health Sciences and Precision Medicine, School of Physics and Electronics, Shandong Normal University, Jinan, China; ^2^Department of Clinical Laboratory, Shandong Provincial Hospital Affiliated to Shandong First Medical University, Jinan, China

**Keywords:** attention mechanism, feature pyramid network, red blood cells, classification, microscopic image

## Abstract

Clinically, red blood cell abnormalities are closely related to tumor diseases, red blood cell diseases, internal medicine, and other diseases. Red blood cell classification is the key to detecting red blood cell abnormalities. Traditional red blood cell classification is done manually by doctors, which requires a lot of manpower produces subjective results. This paper proposes an Attention-based Residual Feature Pyramid Network (ARFPN) to classify 14 types of red blood cells to assist the diagnosis of related diseases. The model performs classification directly on the entire red blood cell image. Meanwhile, a spatial attention mechanism and channel attention mechanism are combined with residual units to improve the expression of category-related features and achieve accurate extraction of features. Besides, the RoI align method is used to reduce the loss of spatial symmetry and improve classification accuracy. Five hundred and eighty eight red blood cell images are used to train and verify the effectiveness of the proposed method. The Channel Attention Residual Feature Pyramid Network (C-ARFPN) model achieves an mAP of 86%; the Channel and Spatial Attention Residual Feature Pyramid Network (CS-ARFPN) model achieves an mAP of 86.9%. The experimental results indicate that our method can classify more red blood cell types and better adapt to the needs of doctors, thus reducing the doctor's time and improving the diagnosis efficiency.

## Introduction

**Figure 1:**
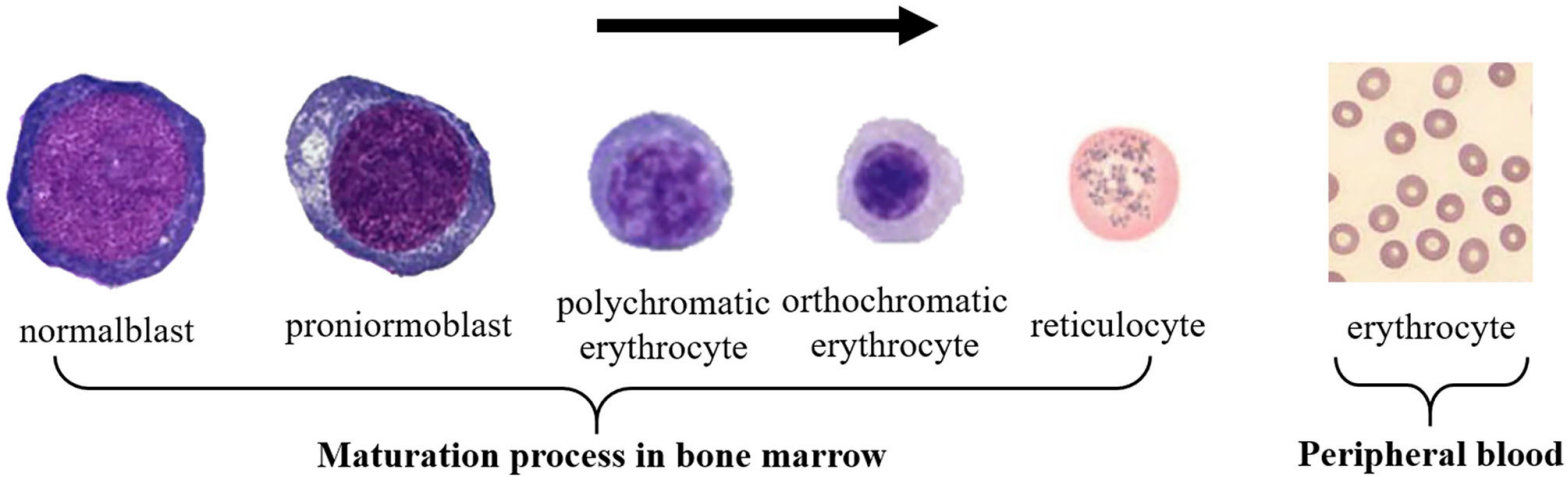
Red blood cell maturation process.

As a connective tissue, blood has the following four forms, namely white blood cells (WBCs), red blood cells (RBCs), platelets, and plasma. Plasma can be regarded as an intercellular substance. The other three types of cells can be distinguished according to their shape, size, presence or absence of nucleus, color, and texture (1). RBCs are the majority component of blood cells, which transport oxygen to various parts of the human body and discharge the carbon dioxide produced by the human body (2, 3). The morphology of RBCs is non-nucleated, with biconvex and concave round pie-shaped cells. Its average diameter and thickness of this type of cell are about 7 and 2.5 μm, respectively. RBCs are produced in the bone marrow, and the development of primitive RBCs into mature RBCs consists of four stages: basophilic normoblast, polychromatic normoblast, orthochromatic normoblast, and reticulocytes. After mature, RBCs enter the peripheral blood, as shown in Figure 1. The average life span of RBCs is about 120 days, and abnormal RBCs may live longer or shorter. Common RBC abnormalities include polycythemia, erythropenia, decreasing or increasing in size and hemoglobin, and changes in RBC morphology.

Diseases associated with RBCs include anemia, malaria, kidney tumors, malnutrition, and hemolytic disease, and anemia is the most common disease (4). These diseases cause many abnormal RBCs to appear in the peripheral blood. Mainly manifested as a change in the shape, size, and hemoglobin content of RBCs (5). Since abnormal RBCs may be a signal of certain diseases (6, 7), the detection and classification of RBCs are of great significance for the timely detection of diseases.

Clinically, doctors need to use a microscope to check whether there are abnormal RBCs or immature cells in the peripheral blood (8). In this case, there are usually hundreds of RBCs in the field of view, and a large number of images are obtained by microscopic image capturing equipment. This requires a lot of manpower. Meanwhile, the operation relies on the subjective judgment of the doctor, and different operators may produce different results (9), which will affect the accuracy of the test results.

In recent years, with the development of image processing technology, medical image analysis has become an indispensable tool in medical research, clinical disease diagnosis, and treatment (8). This technique has been used to analyze various types of medical images and extract more useful medical information from images to help clinical diagnosis. An automatic and effective cell classification method can be used to assist doctors in improving treatment plans and predicting treatment results. At present, the microscopic images generally have the following shortcomings: (1) The image capture process is affected by many factors such as light, color changes, blurring, etc.; (2) There may be interferences such as noise. In recent years, deep learning has developed into a research hotspot in medical image analysis. It can extract the hidden diagnosis features from medical images and solves the problems in medical image processing, such as object tracking (10), multi-label classification (11), pedestrian detection (12), and multi-class classification (13). Aiming at the challenges in RBC images, our study attempts to use the deep learning method to greatly improve the efficiency of doctors and ensure the accuracy and objectivity of the detection results.

A lot of research works have been done on the detection and classification of RBCs. Yi et al. (14) proposed a method to analyze the equality of the covariance matrix in the Gabor filtered holographic image to automatically select the linear or non-linear classifier for RBC classification. This method used a single RBC image to classify three types of RBCs. Maji et al. (15) proposed to use mathematical morphology to automatically characterize RBCs. Mahmood et al. (16) used geometric features and Hough transform to detect the center of RBCs. Morphology was used to identify and extract RBCs from the background or other cells, Hough transform is used to identify the shape of RBCs. Besides, K-means clustering (17), boundary descriptors (18), and geometric features (19) were used to extract features. Sen et al. (20) used machine learning to divide RBCs into three categories. The method first divides RBCs into individual cells and then extracts features and classifications, which achieves an accuracy of 92%.

Lee et al. (21) proposed a hybrid neural network structure that combines parallel and cascading topologies for RBC classification. The authors used a single RBC image to extract shape features and clustering features. Then, the extracted features were input into a feedforward neural network with a three-layer structure for classification. Jambhekar et al. (22) studied the use of artificial neural networks to classify blood cells. The three-layer network achieves an accuracy of 81% for classifying sickle RBCs, WBCs, and overlapping cells. Elsalamony et al. (23) proposed to use a three-layer neural network to classify sickle cells and elliptical cells using the shape features of RBC. Xu et al. (24) used deep convolutional neural networks to classify eight types of RBCs, and the proposed method achieves an accuracy of 87.5%. Alzubaidi et al. (25) proposed a convolutional neural network using the ECOC model as a classifier. The method divides RBCs into normal cells, sickle cells, and other three categories, which achieves an accuracy of 88.11%. Kihm et al. (26) used a regression-based convolutional neural network to classify two types of RBCs (“slipper” and “croissant”) in a flowing state. Parab et al. (27) used a convolutional neural network to extract and classify individual RBCs after segmentation. They divided RBCs into nine categories and achieved an accuracy of 98.5%. Lin et al. (28) used FPN-ResNet-101 and Mask RCNN to classify two types of RBCs (hRBCs and tRBCs) in quantitative phase images, with an accuracy of 97%.

Most of the current works to segment red blood cell images into individual RBCs and then perform feature extraction and classification; very few works perform direct classification of the entire red blood cell image, and the number of RBCs in each image is small (about dozens). After fully understanding the needs of doctors and summarizing the methods in the research field, this paper proposed an Attention Residual Feature Pyramid Network (ARFPN). In this method, dense red blood cell images (each image contains about 230 red blood cells) are used for direct classification. Meanwhile, the feature pyramid network (29) is combined with spatial and channel attention mechanisms to focus on the multi-scale features related to categories, thus improving the expression of related features and suppressing background features. Besides, an anchor intensive strategy is adopted to better cover RBCs in the proposal stage. Moreover, the RoI align method is used to improve the extraction accuracy of RoI and locate the object more accurately. The contributions of this paper are summarized as follows: (i) the method can detect and classify 14 red blood cells; (ii) there is no single red blood cell segmentation, which simplifies the implementation steps and improves the efficiency; (iii) the method provides convenience for doctors, which can better adapt to the needs of doctors, and has better clinical applicability.

The rest of this paper is organized as follows. Section Materials and Methods introduces the used data set, data preprocessing methods, and the feature extraction and classification methods based on the channel and spatial attention feature pyramid network; section Results analyzes and introduced the experimental results; the results are discussed in section Discussion. Finally, conclusions are put forward in section Conclusions.

## Materials and Methods

**Figure 2:**
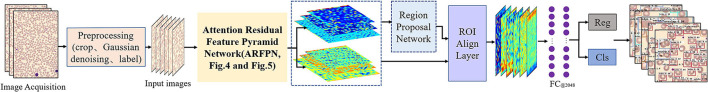
RBC classification based on the ARFPN classification network.

As shown in Figure 2, the workflow of our proposed method for RBC classification includes the image processing stage, feature extraction stage, post-processing stage, and cell classification stage. Each stage is described in detail in the following.

### Data Acquisition

**Figure 3:**
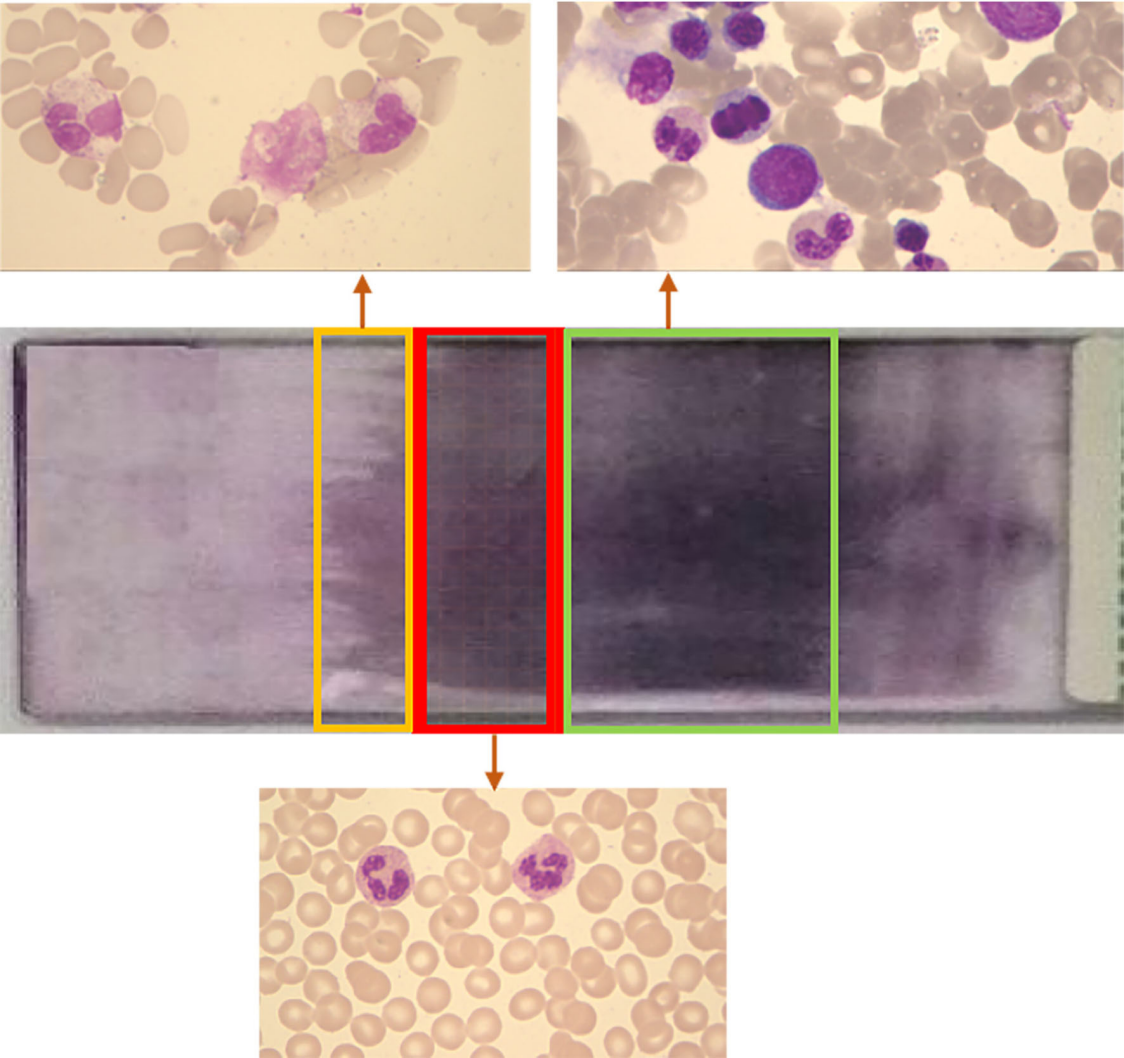
Schematic diagram of blood smear. A large number of red blood cells overlap in the green box area. The number of red blood cells in the yellow frame area is small. The number of cells in the red frame area is appropriate and evenly distributed, which is suitable for observation.

The dataset was collected from the Department of Clinical Laboratory of Shandong Provincial Hospital, affiliated with Shandong First Medical University. The RBC images in the dataset were collected by CellaVision DM96 (CellaVision AB, Lund, Sweden). The blood sample was put into a blood smear and then detected by the device to capture the image. The finished blood smear is shown in Figure 3.

The resolution of each collected images is about 1,418 × 1,420. There are approximately 1,300 RBCs in each image (not including edge cells). All collected microscope images are in BMP format and contain RGB channels. Three types of cells are included in the images, i.e., RBC, white blood cells (WBC), and platelets. The obtained data set was verified by experienced doctors to avoid the interference of external factors, such as light.

### Pre-processing

The obtained images were preprocessed to make them more suitable for our study. First, the image in BMP format was converted to JPG format, and the noise was eliminated by a Gaussian filter. Then, the image was cropped according to the Pascal VOC dataset format. The size of the cropped image is 375 × 500, and the number of RBCs in the image is usually more than 200. The repeat parameter was set to 30% during cropping to expand the data. In the cropping process, the image containing many rare RBCs was horizontally flipped to expand the data and increase the sample size. Finally, LabelImg was adapted to label the RBCs in the image. Labeling and inspection were conducted by two experienced doctors. All the RBCs were divided into 14 categories (schistocyte, spherocyte, stomatocytes, target cells, hypochromic, elliptocytes, normal RBCs, overlapping RBCs, hyperchromic, microcyte, macrocyte, teardrop cells, basophilicstippling and the cells at the edge of the image). In RBC image, the resolution size of normal RBCs is 21 × 21, those with a resolution >24 × 24 are macrocyte, and those with a resolution <18 × 18 are microcyte. The schistocytes are broken red blood cells that resemble “fragments” in shape. Hyperchromic and hypochromic are related to the content of hemoglobin, and elliptocytes are shaped like ellipses. The target cell is shaped like a “shooting target,” and stomatocytes is shaped like a “mouth.” The corresponding quantity of each RBC category is listed in Table 1. The obtained dataset was used to evaluate our method and compare the results. After preprocessing, there are 588 images in total, each of which is a 350 × 500 × 3 RGB image. Four hundred and seventy images were used as the training set, and the remaining 118 images were used as the test set. As shown in Table 2.

**Table 1:** Various types of RBCs.

**Name**	**Number**	**Name**	**Number**
Schistocyte	772	Overlap	6,064
Spherocyte	4,021	Hyperchromic	17,693
Stomatocytes	615	Microcyte	17,060
Target cells	537	Macrocyte	9,084
Hypochromic	5,414	Teardrop cells	1,287
Elliptocytes	15,439	Basophilicstippling	451
Normal RBC	18,126	Edge cells	20,661

**Table 2:** Allocation of training set and test set (number of cells and number of images).

	**Cell number**	**Image number**
Train	9,3885	470
Test	2,3366	118

### Feature Extraction of Shape, Size, and Hemoglobin Content

The size of normal RBCs is about 7~8 μm, which is reflected in the image with a resolution of 21 × 21. The size of abnormal RBCs in the image varies widely, and each has its specific shape. The characteristics of the RBC images can be summarized as: (a) Large changes in cell size; (b) RBCs are small objects; (c) Cells are densely distributed; (d) The contrast between the RBC and the background is low. In deep learning object detection, the detection of small objects has always been a difficult problem due to low resolution, blurry pictures, less information, and weak feature expression. This study used feature pyramid network (FPN) to overcome the above problems because it can better deal with the multi-scale changes in object detection. The FPN makes reasonable use of the features of each layer in the convolutional network and merges the features of different layers. Specifically, it constructs a top-down, horizontally connected hierarchical structure that combines low-resolution and strong semantic features with high-resolution and weak semantic features.

In recent years, attention network models have achieved good performance in classification tasks. In this research, channel attention mechanism (30) and spatial attention mechanism (31) were integrated into the feature extraction network to achieve accurate classification of RBCs. In the feature extraction stage, the attention mechanism (32) can highlight the features related to categories while focusing on the key features of red blood cells and generating more discriminative feature representations. The integration of these two mechanisms contributes to a great performance improvement when the number of growth parameters is small.

#### Channel Attention Residual Feature Pyramid Network

**Figure 4:**
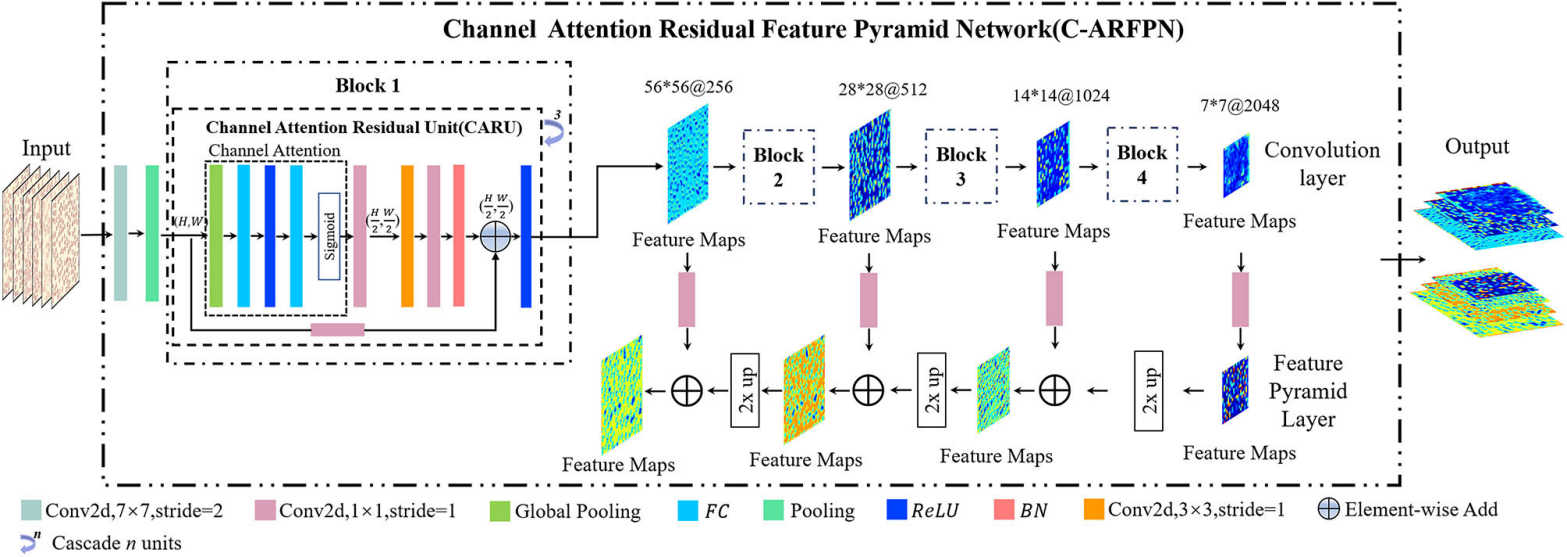
Schematic diagram of the Channel Attention Residual Feature Pyramid Network (C-ARFPN) structure. Resnet-101 is used as the backbone. Channel Attention Residual Units (CARU) are located at the front of each residual congestion unit and stacked in varying numbers to form residual blocks.

The structure of Channel Attention Residual Feature Pyramid Network (C-ARFPN) is shown in Figure 4. ResNet-101 and ResNet-50 are used as the backbone of our network. Each bottleneck of the residual network is replaced with channel attention residual units (CARUs) that are located behind the residual unit. CARU first averages and pools the input features, so that the features can respond to the global distribution, thus expanding the global receptive field and reducing the calculation amount. The following two full connection layers map the channel feature representation to the sample label space, and the output represents the weight of each feature channel. The feature channel is weighted with the input feature to recalibration the input feature on the channel dimension.

#### Channel and Spatial Attention Residual Feature Pyramid Network

**Figure 5:**
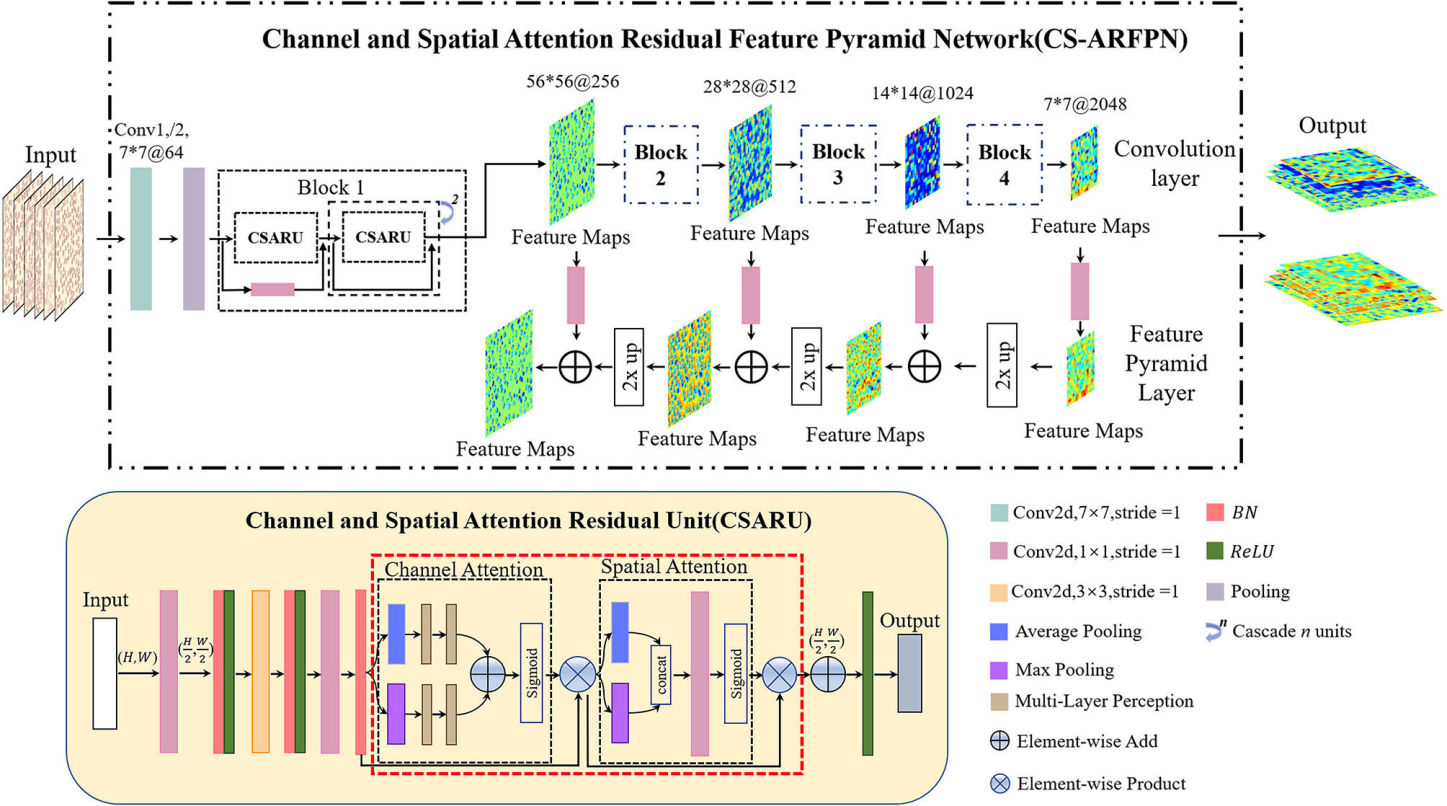
Schematic diagram of the Channel and Spatial Attention Residual Feature Pyramid Network (CS-ARFPN) structure. This structure still uses ResNet-101 as the backbone. Channel and Spatial Attention Residual Units (CSAU) are located behind each residual congestion unit. They focus on the key and detailed features of the red blood cell image.

The structure of Channel and Spatial Attention Residuals Feature Pyramid Net-work (CS-ARFPN) is shown in Figure 5. The upper part of the figure illustrates the overall flow chart of feature extraction. Similarly, ResNet-50 and ResNet-101 are used as the backbone. The lower part of the figure shows the structure of the Channel and Spatial Attention Residual Unit (CSARU), which is nested in each residual unit of the residual network and located behind the three convolution cores. The input feature is first compressed in the spatial dimension, and average pooling and maximum pooling are used to aggregate the spatial information of the feature map. The obtained feature space information is sent to the multi-layer perceptron for element-by-element summation, and the compressed space information is multiplied by the original feature points to obtain the channel attention feature. Then, the channel attention feature is input to the spatial attention unit, and average pooling and max pooling are used to compress channels and extract the maximum value. After dimensionality reduction through convolution operation, the attention feature is obtained by dot product with the original channel attention feature.

Both C-ARFPN and CS-ARFPN use ResNet-101 and ResNet-50 as the backbone. Each attention module is distributed in a residual unit according to its position. Since RBCs are small objects, the feature pyramid network combined with the attention mechanism can merge deep and shallow features and focus on category-related features. This improvement makes the characteristics of RBCs more accurate and richer, thus improving the model's detection and classification ability of small objects, and improving the performance of the model.

### Post-processing and Classification

After feature extraction, the features are input to the subsequent network for post-processing and classification. First, the feature map is input into the RPN to filter out the anchors containing the foreground. Then, the high-quality object candidate box is selected and input into the ROI pooling layer. In the pooling operation, RoI align (33) instead of RoI pooling operation is used. Compared with RoI pooling, RoI align removes the quantization rounding operation, so it can overcome the bounding box offset problem (34) and extract more accurate RoI. After the RoI align operation is performed on the feature map, the candidate recognition regions of different sizes are normalized into a fixed-size object recognition region.

**Figure 6:**
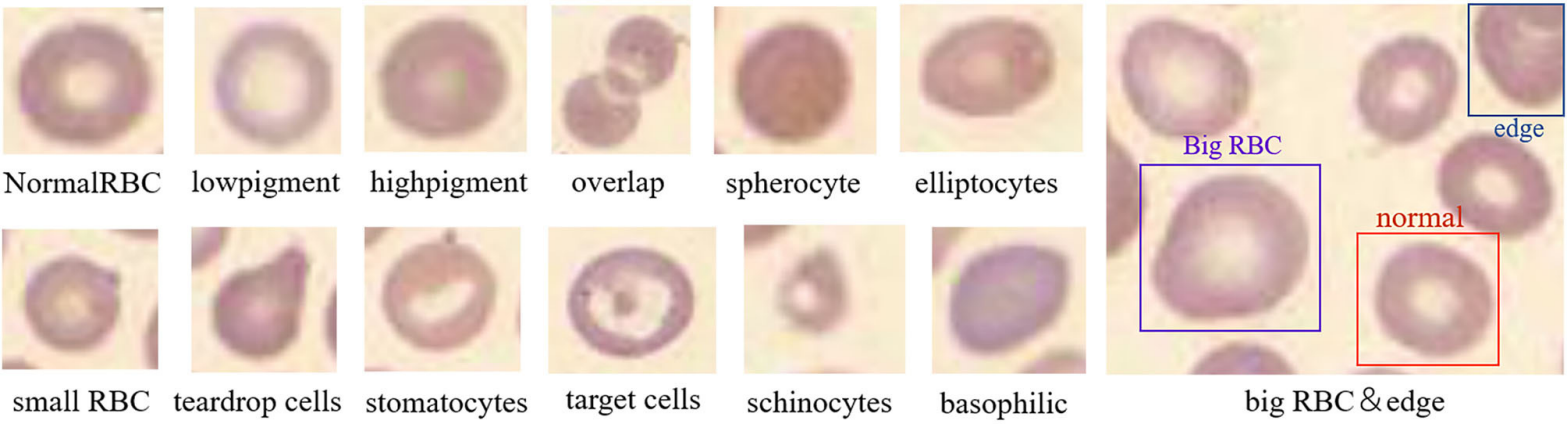
Schematic diagram of normal and abnormal red blood cells. This picture illustrates the most obvious characteristics of each red blood cell, such as the shape, size, and hemoglobin content.

The features after RPN and RoI pooling are sent to the subsequent network for classification and regression. In this process, 14 types of RBCs are classified including schistocyte, spherocyte, stomatocytes, target cells, hypochromic, elliptocytes, normal RBCs, overlapping RBCs, hyperchromic, microcyte, macrocyte, teardrop cells, basophilic stippling, and the cells at the edge of the image. The schematic diagram is shown in Figure 6. Normal RBCs and macrocytes are displayed in one image to make their difference obvious. It can be seen from the figure that each cell has its characteristics. During the training process, the weight of the network is adjusted according to the input data to minimize the error between the input and the target. Then Fast RCNN (35) is used to perform cell classification, and the output of the classification prediction is converted into a probability distribution through softmax.

### Ablation Study

Our proposed method uses the attention module to learn the features related to categories. First, the effectiveness of the attention module was verified, and the performance of two different attention modules was compared. Meanwhile, the performance of RoI align and RoI pooling methods was compared. Besides, the impact of Adam optimizer, momentum optimizer, and various training parameters on the model performance was investigated. All comparisons and analyses were performed under the same parameter settings. Moreover, the effectiveness of the proposed model on different was verified public datasets.

### Training Implementation

Our proposed method was implemented on a computer equipped with Intel^®^ Core™ i7-8700k CPU@3.70GHz with 32GB memory, and the computationally intensive calculations were offloaded to an Nvidia Tesla P100 GPU with 16 GB HBM2 memory and 3,584 computer unified device architecture (CUDA) cores. To visually present the obtained model parameters, all experiments were conducted using Python programming language under the TensorFlow framework (36). In the training process, momentum and Adam optimizer were used in the parameter configuration to minimize the loss. The batch size was set to 1, and the number of iterations was set to 110,000. It takes 35 h to complete the optimization. In the early stage of training, a large learning rate was used to make the model easy to obtain the optimal solution; in the later stage of training, a small learning rate was used to ensure that the model will not fluctuate too much. The learning rate was divided by 10 after 60,000 and 80,000 iterations, and the minimum learning rate was set to 10^−6^. Besides, the momentum of the model was set to 0.9, and the weight decay was set to 10^−4^. During training, random initialization was used to initialize the weights, and used the cross-entropy loss function was adopted to evaluate the error between the predicted value and the true value of our model. The calculation formula of the cross-entropy loss function is shown in Equation (1).


(1) $$\begin{array}{r} C=\overset{k}{\underset{i=1}{\sum }}y_{i}\;log\left(p_{i}\right) \end{array}$$

where *k* represents the number of classes; *y*_*i*_ represents the label of category *i*; *p*_*i*_ represents the output probability of class *i*, and this value was calculated by Softmax.

### Evaluation Metrics

In our experiments, the metrics of precision, recall, and F1-score were taken to evaluate the performance of our proposed method. The calculation formulas of the evaluation metrics are expressed in Equations (2–4).


(2) $$\begin{array}{rcl} Precsion & = & \frac{TP}{TP+FP} \end{array}$$

(3) $$\begin{array}{rcl} Recall & = & \frac{TP}{TP+FN} \end{array}$$

(4) $$\begin{array}{rcl} F1\;score & = & \frac{2\;\times Precision\;\times \;Recall}{Precision\;+\;Recall} \end{array}$$

Where, TF (True Positive) indicates the number of positive samples that are also judged by the model as positive; TN (True Negative) indicates the number of negative samples that are also judged by the model as negative; FN (False Negative) indicates number of positive samples that are judged by the model as negative. FP (False Positive) indicates the number of negative samples that are judged by the model as positive. Based on this, precision is the ratio of the number of correctly predicted positive examples to the number of samples predicted as positive; recall is the ratio of correctly predicted positive examples to the number of real positive samples. F1 score is the harmonic average of precision and recall, so it can comprehensively reflect the performance. In general, the higher the F1 score, the better the performance of the model.

## Results

### Ablation Study: Comparison of FPN With or Without Attention Module, ROI Pooling or ROI Align, and Others

In the ablation study, ResNet-101 and ResNet-50 were used as the backbone in the training, and different learning rates were set. Since the RBC object is small and densely distributed, a small anchor size was used.

In Table 3, FPN is the original model without attention module; C-ARFPN is the feature pyramid network with channel attention residual unit; CS-ARFPN is the feature pyramid network with channel and spatial attention residual unit. It can be seen that the CS-ARFPN model achieves better performance. Compared with FPN, the accuracy, recall, F1 score, and mAP of CS-ARFPN and S-ARFPN are improved by {4.4, 7.9, 5.7, 6.1%} and {5.5, 7.4, 6.0, 7.2%}, respectively. Compared with the S-ARFPN model, the accuracy, recall, F1 score, and mAP of the CS-ARFPN model is improved by 1.1, −0.7, 0.3, and 0.9%.

**Table 3:** The evaluation metrics of the model with/without the attention module. The best results are shown for each model.

**Models**	**Recall**	**Precision**	**F1**	**mAP**
FPN	0.756	0.759	0.759	0.798
Our proposed (S-ARFPN)	0.8	0.838	0.816	0.86
Our proposed (CS-ARFPN)	0.811	0.831	0.819	0.869

**Figure 7:**
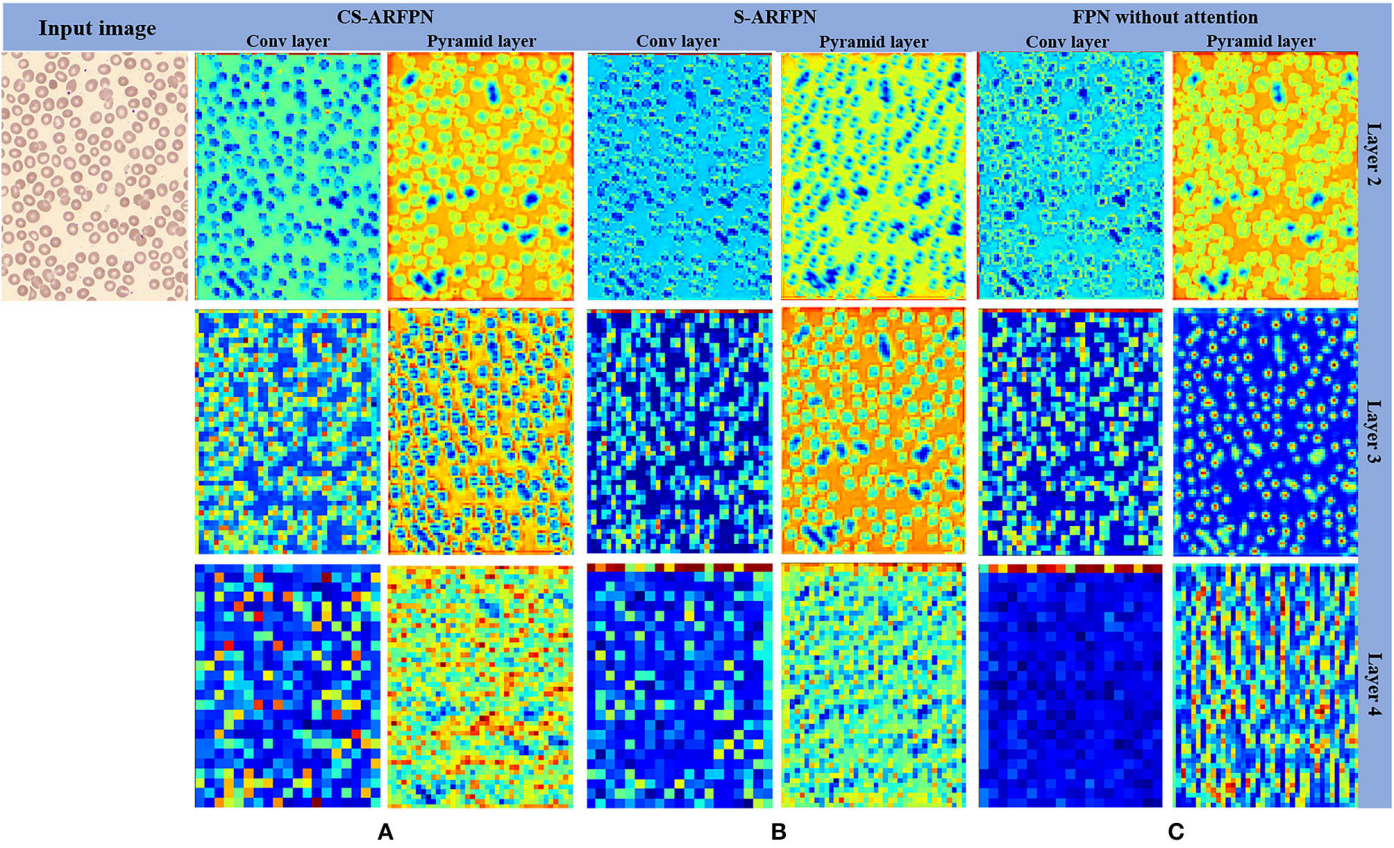
**(A)** The feature map of the Channel Spatial Attention Residual Feature Pyramid Network (CS-ARFPN) model; **(B)** The feature map of the Spatial Attention Residual Feature Pyramid Network (S-ARFPN) model; **(C)** The feature map of the original FPN model. The feature maps extracted from each layer are presented, where warmer colors, such as red and yellow, indicate higher attention weights. The figure, the model with an attention module has a stronger expression of target characteristics and focuses more on the object.

Figure 7 presents the feature map of the two models with different attention residual units and the original FPN model. The leftmost column shows the input image, and the next six columns show the feature maps of the three models including CS-ARFPN, S-ARFPN, and FPN (each model contains two columns of feature maps). They are the feature maps extracted by the convolutional layer {C2, C3, C4} and the pyramid layer {P2, P3, P4}. It can be seen that the feature maps extracted by the CS-ARFPN model pay more attention to the object to be recognized, so the model achieves better performance.

**Figure 8:**
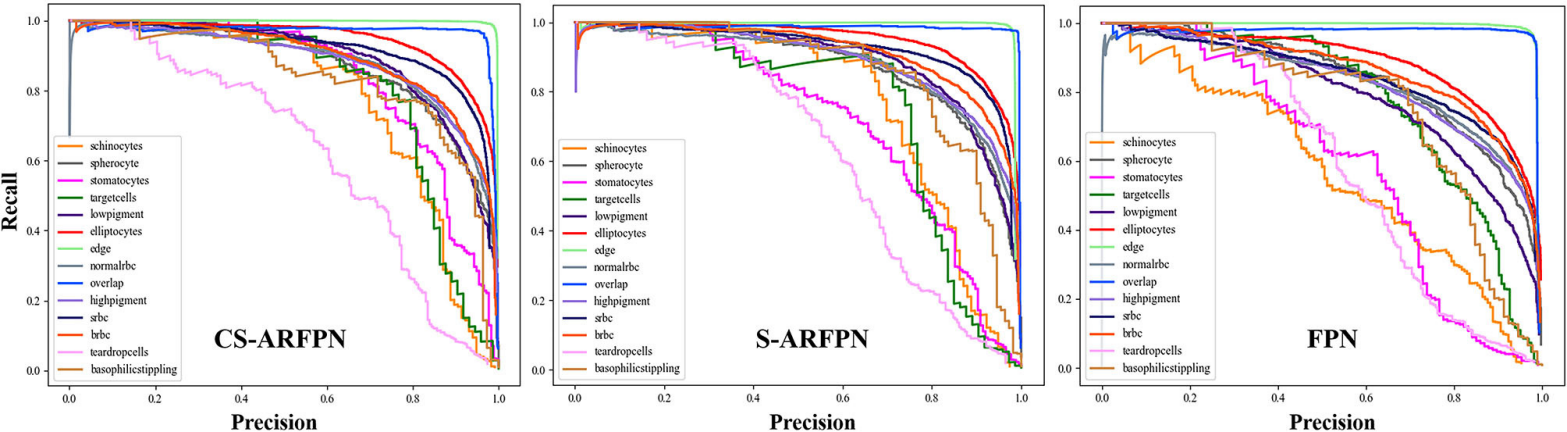
Precision-Recall (PR) curve of the cell classification results. Different colored PR curves represent different types of RBCs. The closer the curved surface is to the upper right, the better the classification effect of the red blood cell.

Figure 8 shows the precision-recall curve (PR) of the three models of FPN, C-ARFPN, and CS-ARFPN. The closer the curve to the upper right, the larger the area under the line and the better the performance of the model. The PR area under the curve (PR-AUC) of the three models is 0.798, 0.86, 0.869, respectively. Thus, the CS-ARFPN model achieves the best performance, followed by C-ARFPN.

Table 4 lists the recall, precision, F1-score, and AP of the CS-ARFPN model for classifying the 14 types of cells.

**Table 4:** Results of different red blood cells by the CS-ARFPN model.

**Class**	**Recall**	**Precision**	**F1**	**AP**
Schistocytes	0.681	0.841	0.753	0.787
Spherocyte	0.822	0.782	0.801	0.879
Stomatocytes	0.697	0.853	0.768	0.840
Target cells	0.744	0.821	0.786	0.815
Hypochromic	0.772	0.855	0.811	0.890
Elliptocytes	0.890	0.859	0.874	0.946
Edge	0.987	0.987	0.987	0.997
Normal RBC	0.804	0.810	0.807	0.880
Overlap	0.966	0.964	0.965	0.973
Hyperchromic	0.838	0.791	0.814	0.883
Microcyte	0.876	0.842	0.859	0.913
Macrocyte	0.857	0.784	0.819	0.893
Teardrop cells	0.570	0.686	0.622	0.631
Basophilic	0.837	0.761	0.797	0.843

Table 5 shows the AP of the three models with two different optimization strategies. In the FPN, C-ARFPN, and CS-ARFPN models, the Momentum optimizer leads to 1.9, 18.7, and 9.7% higher performance than the Adam optimizer, respectively.

**Table 5:** Performance comparison of models using Adam and Momentum.

	**FPN**	**S-ARFPN**	**CS-ARFPN**
Adam	0.779	0.673	0.774
Momentum	0.798	0.860	0.869

The performance of using two different RoI processing methods, i.e., Roi pooling and RoI align is shown in Table 6. The model using the RoI align method achieves better performance than that using the RoI pooling method.

**Table 6:** Comparison of the precision of ROI pooling and ROI align methods.

	**FPN**	**S-ARFPN**	**CS-ARFPN**
ROI Pooling	0.786	0.823	0.858
ROI Align	0.798	0.860	0.869

### Comparison With the State-of-the-Art Models and Comparison of Results Obtained on Other Data Sets

Our proposed method was compared with five classification methods based on deep learning, including Faster RCNN (32), RetinaNet (37), Cascade RCNN (38), R-FCN (39), and Cascade-FPN (40). All the models used were trained from scratch on the RBC dataset.

ResNet-50 and ResNet-101 were used as the backbone for model training; then, different parameters were set to finetune the models; finally, showed the best results for each model were obtained. Table 7 lists the classification performance of different models. The highest performance values are bolded in the table. The mAP of the two proposed models is 0.86 and 0.869, respectively, and the two models achieve the best performance among all the models.

**Table 7:** Comparison of our proposed method with other advanced methods.

**Backbone**	**Models**	**Recall**	**Precision**	**F1**	**mAP**	**Param (MB)**
ResNet-50	Cascade RCNN (33)	0.361	0.687	0.463	0.385	254.47
	Faster RCNN (32)	0.398	0.652	0.481	0.394	54.07
	R-FCN (34)	0.530	0.757	0.638	0.551	95
	RetinaNet (31)	0.695	0.751	0.736	0.684	61.92
	Cascade-FPN (35)	0.759	0.757	0.736	0.736	98.14
	FPN	0.753	0.758	0.759	0.796	79.05
ResNet-101	Cascade RCNN (33)	0.429	0.699	0.525	0.416	290.69
	Faster RCNN (28)	0.447	0.687	0.526	0.434	63.70
	R-FCN (34)	0.528	0.880	0.620	0.548	95.66
	RetinaNet (31)	0.686	0.738	0.709	0.683	133.58
	FPN	0.756	0.759	0.759	0.798	115.27
ResNet-50	Our proposed (S-ARFPN)	0.754	0.758	0.753	0.792	88.67
	Our proposed (CS-ARFPN)	**0.811**	**0.831**	**0.819**	**0.869**	88.68
ResNet-101	Our proposed (S-ARFPN)	**0.800**	**0.838**	**0.816**	**0.860**	133.44
	Our proposed (CS-ARFPN)	0.791	0.789	0.788	0.833	133.43

*Each model was trained under different parameter settings, and the best results of each model are shown. The classification effect of Cascade-FPN is not ideal when the backbone is Resnet101, so it is not displayed in the table. The best results are highlighted in bold*.

Besides, to verify the effectiveness of the proposed model, the performance of our proposed model on different datasets was compared, and the comparison results are listed in Table 8. Among them, in the IDB data set, the accuracy of circular and elongated red blood cells are 99 and 94.4%, respectively. In the BCCD data set, the accuracy of WBC and Platelets are 97.43 and 92.89%, respectively. The proposed model achieves an mAP of 91% and 98.8% on the BCCD dataset and IDB dataset is 91.23%, respectively.

**Table 8:** Comparison of the accuracy of the proposed method on different datasets.

**Dataset**	**Images**	**Class**	**AP (%)**
BCCD (41)	364	WBC	97.43
		RBC	83.05
		Platelets	92.89
IDB (42)	626	circular	99
		elongated	94.4
		other	80.3

## Discussion

To better assist doctors in diagnosing the diseases related to RBCs, this paper proposed an attention feature pyramid network model that can directly classify dense red blood cell images. Since RBCs are small objects, this paper combined the attention mechanism with the feature pyramid network to improve the detection of small objects. The experimental results show that the two proposed attention residual units can capture more key feature information of RBCs, which helps to classify RBCs more accurately.

In the training process, different backbones, learning strategies, and anchor settings were used, and the optimal parameter setting of the two models was obtained after a lot of training. The results show that different learning rates, anchor sizes, backbones, and attention modules led to performance differences. When ResNet-50 was used as the backbone, the CS-ARFPN model achieved the best performance under the learning rate of 0.001 and the anchor size of 32. When ResNet-50 was used as the backbone, the S-ARFPN model achieved the best performance under the learning rate of 0.002 and the anchor size of 4. The subsequent experiment and analysis were conducted based on the model and the above-mentioned optimal parameters.

In the experiment, the performance of FPN, C-ARFPN and CS-ARFPN with two different attention residual unit models was compared to verify the effectiveness of the attention mechanism. The effectiveness of our method was proved through evaluation metrics, feature maps, and PR curves.

It can be seen from Table 3 that, compared with FPN, both CS-ARFPN and C-ARFPN achieved improved performance, which shows the effectiveness of the attention mechanism. Meanwhile, the CS-ARFPN model performed better than the C-ARFPN model, indicating that the CS-ARFPN model pays more attention to features, channel feature information and spatial feature information. To make the function of the attention module more intuitive, the feature maps of FPN, C-ARFPN, and CS-ARFPN are shown Figure 7. It can be seen that as the number of layers increases, the extracted features become more and more abstract and more difficult to understand. Compared with FPN, C-ARFPN and CS-ARFPN weaken the background characteristics and highlight the relevant components of the category, so the two models can more accurately capture the shape and size of RBCs. Besides, CS-ARFPN can better focus on the detailed features than C-ARFPN.

Figure 8 shows the PR curves of the three models. The curves in the figure show that for each type of RBC, different attention modules led to different classification performances. In general, the PR curve obtained by the CS-ARFPN model is located on the upper right, which indicates that the area under the line is the largest, and the performance is the best.

The performance of the Adam optimizer and momentum optimizer was compared and analyzed. The results in Table 5 show that the Momentum optimizer performs better for RBCs classification. During the training process, the Momentum optimizer had a slower convergence speed than the Adam optimizer, but it obtained better results and the generalization performance in our work.

As shown in Table 6, the RoI align method achieved higher AP than the RoI pooling method. The rounding operation in the RoI pooling method has little impact on the classification of large objects, but it will have a huge impact on the classification of small objects such as RBCs. The RoI align method removes the rounding operation, so it can accurately extract RoI and achieve better performance.

Table 4 shows the performance metrics of classifying the 14 types of RBCs obtained by the CS-ARFPN model. Among them, the classification results of teardrop cells and schistocytes are not as accurate as other types. Although the attention mechanism focuses on the features related to categories, inaccurate classifications are caused by certain features. This is because different types of feature extraction have different difficulties, and certain red blood cell types have a specific definition standard. In this case, the model fails to learn the abnormal RBC, thus resulting in misclassifications. Secondly, some types of red blood cell samples are small. Although the RBCs were expanded during the cutting process, the sample imbalance problem still existed in the study, which makes the model fail to learn the characteristics of red blood cells with few samples.

The comparison between our method and other advanced methods is shown in Table 7. Our method achieves better performance than other models. Meanwhile, our method was compared with other red blood cell classification methods, including Kihm et al. (26), Parab et al. (27), Lin et al. (28), and others. These methods all use a single red blood cell image for feature extraction and achieve good accuracy. The classification of the entire red blood cell image can be regarded as the classification of dense small objects with weak feature expression and diverse target changes, so feature extraction is more difficult. Due to this, our method obtains a slightly lower accuracy than the comparison methods. To better compare with other methods and verify the effectiveness and generalization of the proposed method, our method was evaluated on two public data sets, i.e., namely the BCCD dataset and the IDB dataset. As shown in Table 8, the classification results of WBCs and Platelets in the BCCD dataset in Table 8 are better. The reason for the low accuracy of RBC classification is that the dataset is mainly provided for WBC classification, and most of the RBCs in the image are overlapping cells. In the IDB dataset, the classification results of circular and elongated are good. The reason for the low accuracy of the RBCs of the other category is that the category contains many small and medium categories, which poses a challenge to the classification. The results indicate that our method is effective and generalizable, and the classification of the entire image can be further improved.

At present, due to the limited dataset and the imbalance of different types of RBC samples, it is difficult to improve the classification performance. After communicating with the doctor, we will collect RBC images under a microscope so that clear images with obvious RBC characteristics can be obtained. In future work, we will collect more data, especially the rare type of RBC. Meanwhile, we will investigate the use of a fully connected layer and loss function of the model to reduce the impact of sample imbalance to further improve the classification performance.

## Conclusions

Abnormal red blood cells can cause changes in shape, size, and amount of hemoglobin, which are closely related to the diagnosis of many diseases. This paper proposed a classification method that can directly classify 14 types of red blood cells on the entire red blood cell image. The feature pyramid network extracts the multi-scale features of RBCs, and the attention mechanism is used to improve the learning and representation of RBC features. Besides, the ROI alignment layer with good performance is used to unify the size of the candidate area. This method proposed in this study can achieve accurate red blood cell classification, which provides a clinically feasible, universal and convenient method for the diagnosis of red blood cell diseases.

## Data Availability Statement

The raw data supporting the conclusions of this article will be made available by the authors, without undue reservation.

## Author Contributions

WS and PH: conceptualization and methodology. JW, YS, and JZ: data production and curation. ZL: data curation, resources, and supervision. WS: validation, writing–original draft preparation, investigation, and visualization. DLi: supervision, project administration, and funding acquisition. DLiu: writing–review and editing and formal analysis. All authors contributed to the article and approved the submitted version.

## Funding

This work was funded by the National Natural Science Foundation of China (61971271), the Taishan Scholars Project of Shandong Province (Tsqn20161023), and the Primary Research and Development Plan of Shandong Province (No. 2018GGX101018, No. 2019QYTPY02).

## Conflict of Interest

The authors declare that the research was conducted in the absence of any commercial or financial relationships that could be construed as a potential conflict of interest.

## Publisher's Note

All claims expressed in this article are solely those of the authors and do not necessarily represent those of their affiliated organizations, or those of the publisher, the editors and the reviewers. Any product that may be evaluated in this article, or claim that may be made by its manufacturer, is not guaranteed or endorsed by the publisher.
