# Supplementary figures and images for: Red Blood Cell Classification Based on Attention Residual Feature Pyramid Network
Source: Front Med (Lausanne). 2021 Dec 14;8:741407. doi: 10.3389/fmed.2021.741407 (PMC8712440; doi:10.3389/fmed.2021.741407)

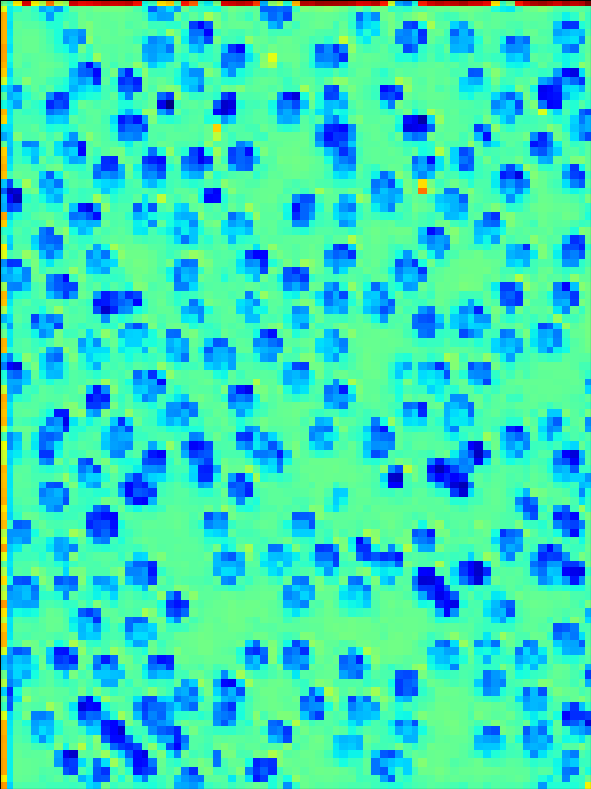

Supplement: Supplementary file 1 [file Data_Sheet_1.zip › Figure7/CS-ARFPN-ResNet50/layer2 conv.png]

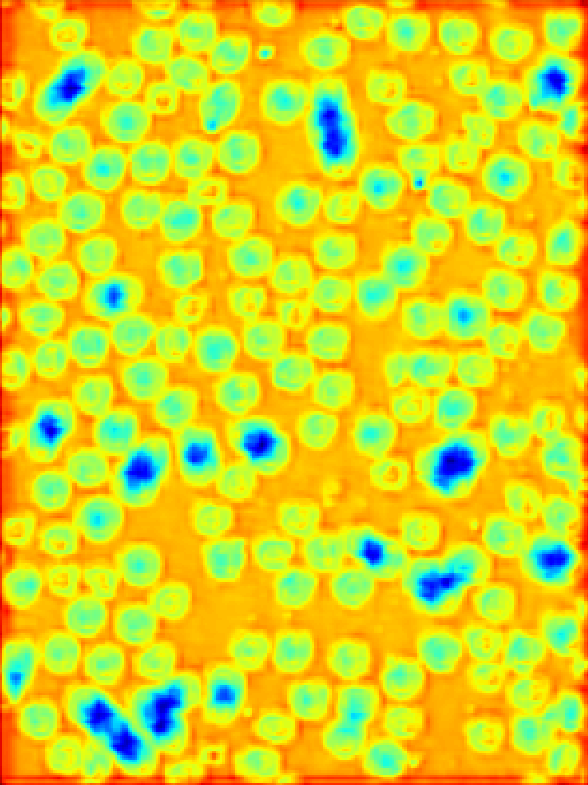

Supplement: Supplementary file 1 [file Data_Sheet_1.zip › Figure7/CS-ARFPN-ResNet50/layer2 pyramid.png]

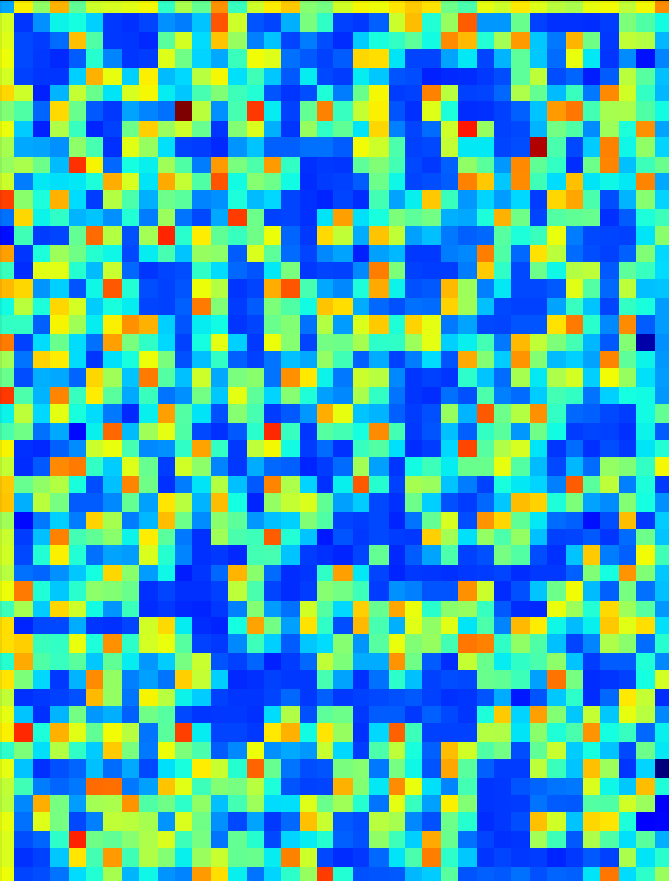

Supplement: Supplementary file 1 [file Data_Sheet_1.zip › Figure7/CS-ARFPN-ResNet50/layer3 conv.png]

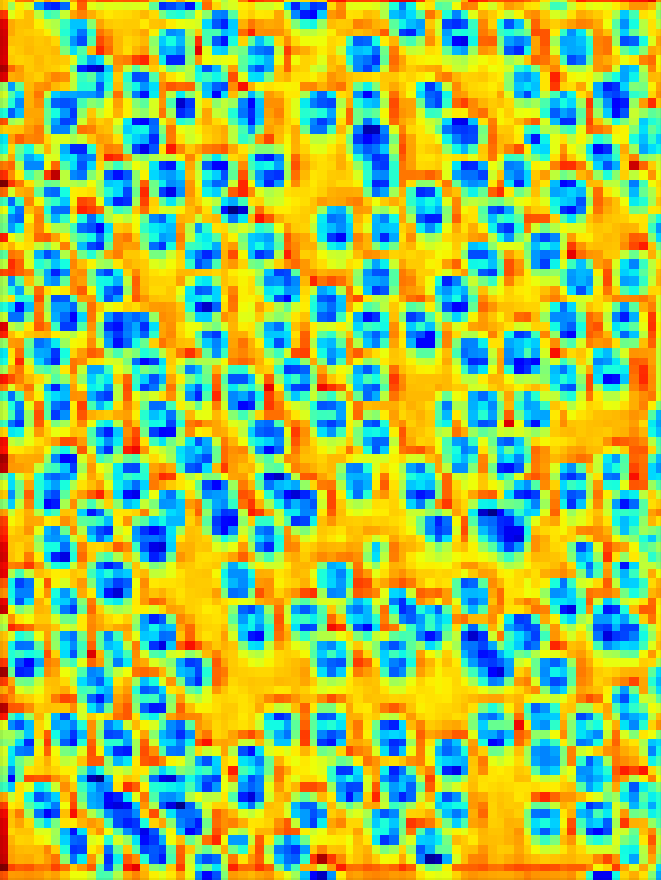

Supplement: Supplementary file 1 [file Data_Sheet_1.zip › Figure7/CS-ARFPN-ResNet50/layer3 pyramid.png]

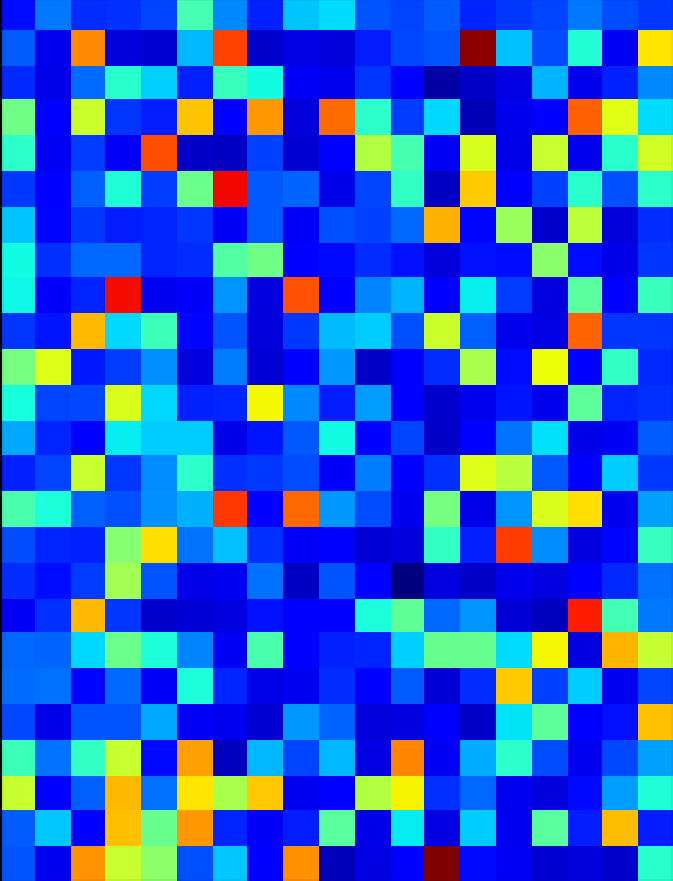

Supplement: Supplementary file 1 [file Data_Sheet_1.zip › Figure7/CS-ARFPN-ResNet50/layer4 conv.png]

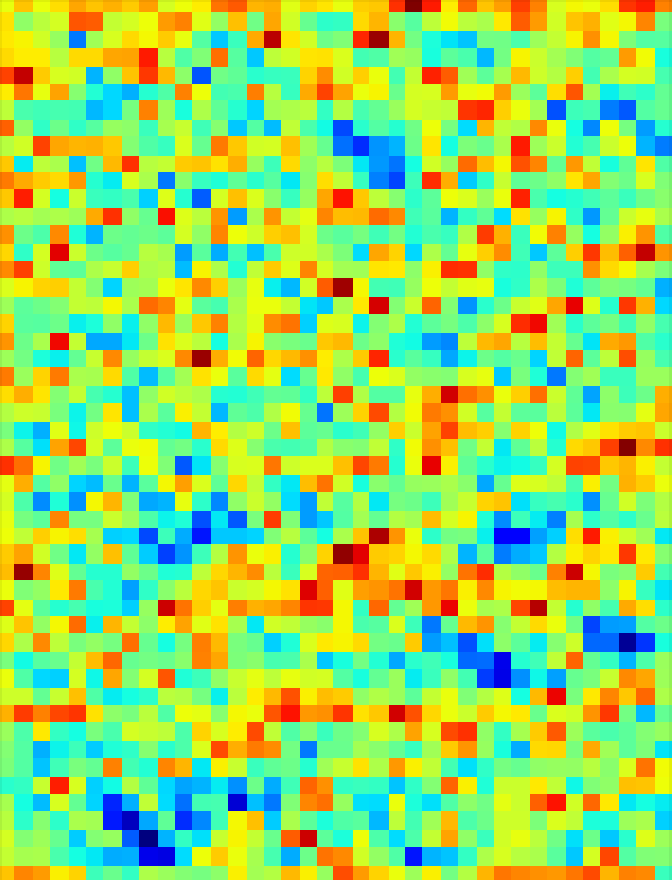

Supplement: Supplementary file 1 [file Data_Sheet_1.zip › Figure7/CS-ARFPN-ResNet50/layer4 pyramid.png]

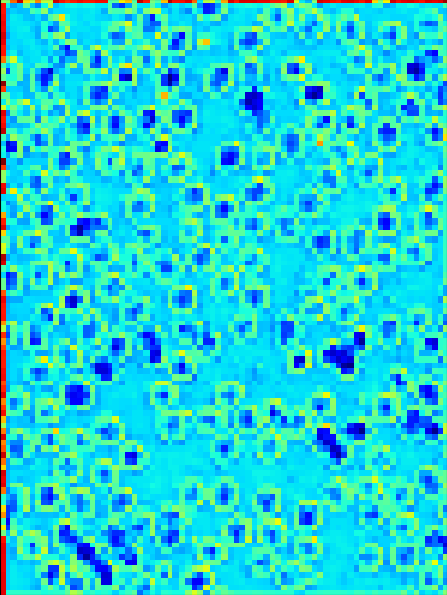

Supplement: Supplementary file 1 [file Data_Sheet_1.zip › Figure7/FPN-ResNet101/layer2 conv.png]

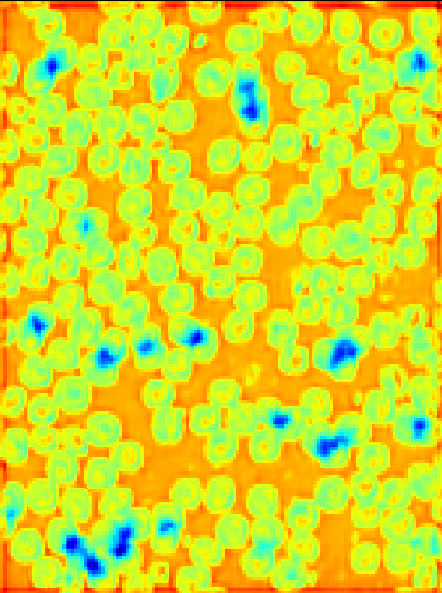

Supplement: Supplementary file 1 [file Data_Sheet_1.zip › Figure7/FPN-ResNet101/layer2 pyramid.png]

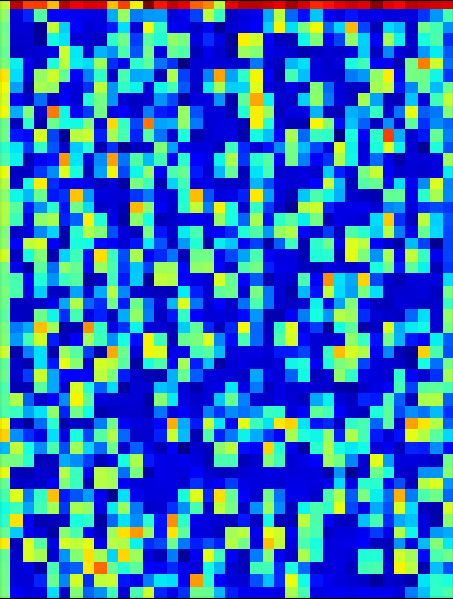

Supplement: Supplementary file 1 [file Data_Sheet_1.zip › Figure7/FPN-ResNet101/layer3 conv.png]

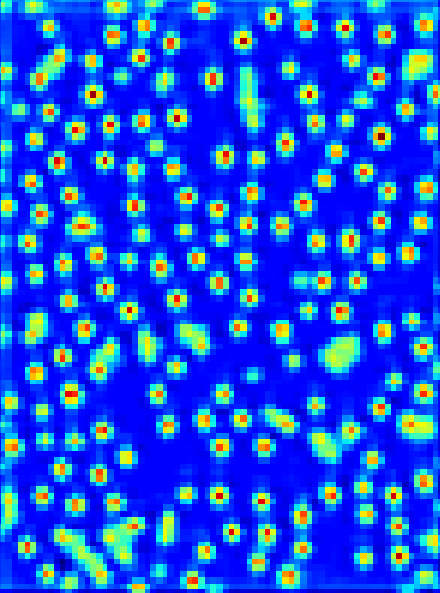

Supplement: Supplementary file 1 [file Data_Sheet_1.zip › Figure7/FPN-ResNet101/layer3 pyramid.png]

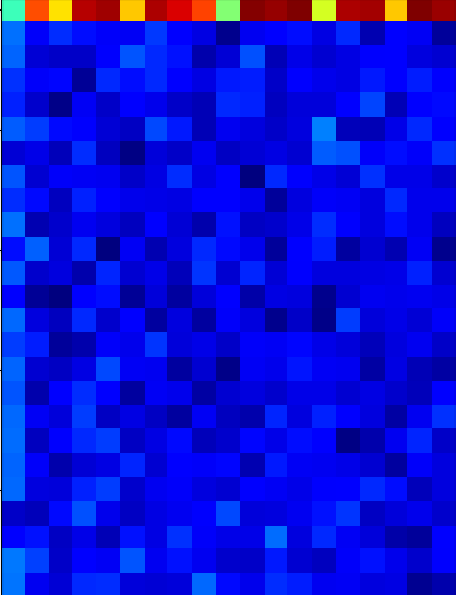

Supplement: Supplementary file 1 [file Data_Sheet_1.zip › Figure7/FPN-ResNet101/layer4 conv.png]

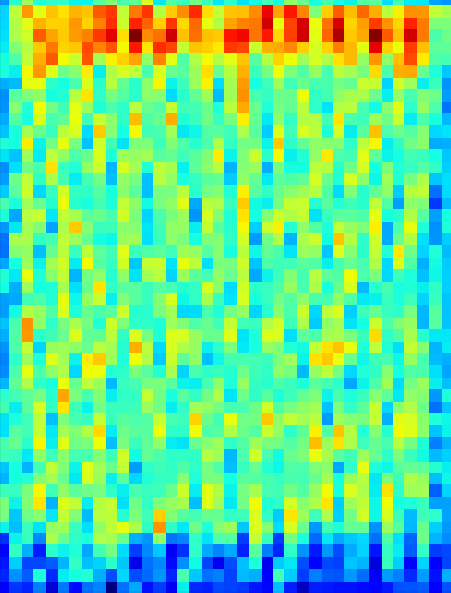

Supplement: Supplementary file 1 [file Data_Sheet_1.zip › Figure7/FPN-ResNet101/layer4 pyramid.png]

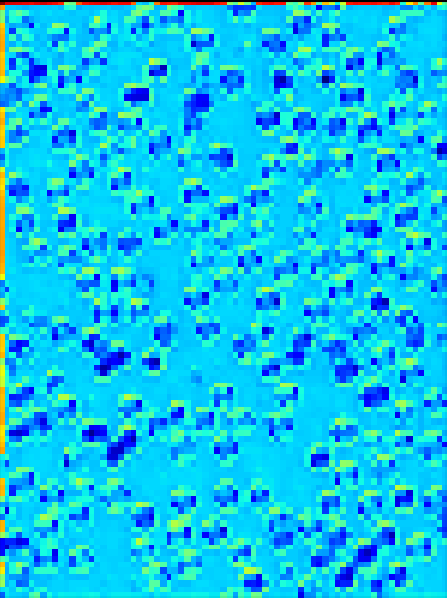

Supplement: Supplementary file 1 [file Data_Sheet_1.zip › Figure7/S-ARFPN-ResNet101/layer2 conv.png]

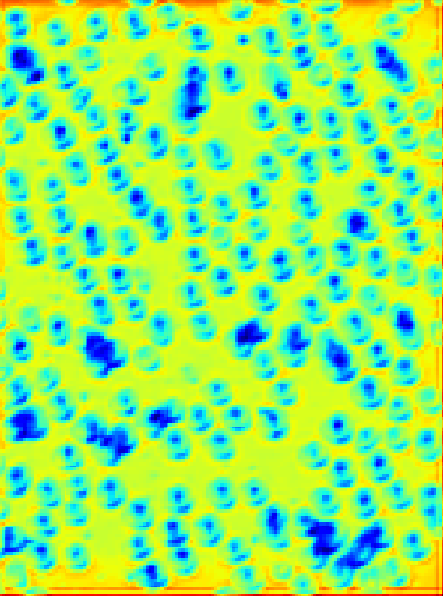

Supplement: Supplementary file 1 [file Data_Sheet_1.zip › Figure7/S-ARFPN-ResNet101/layer2 pyramid.png]

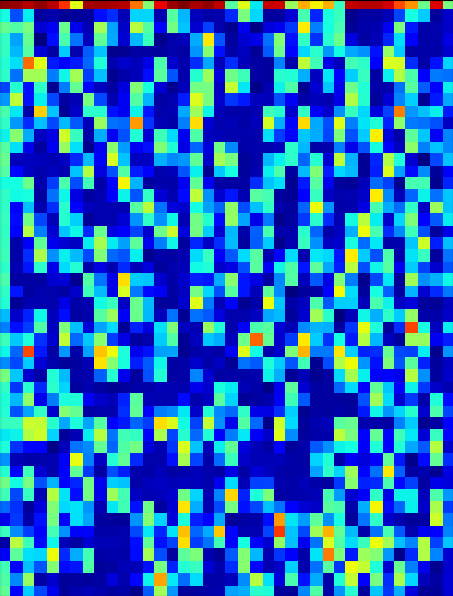

Supplement: Supplementary file 1 [file Data_Sheet_1.zip › Figure7/S-ARFPN-ResNet101/layer3 conv.png]

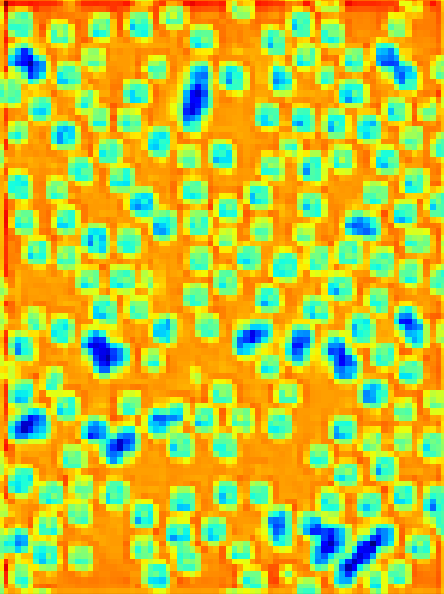

Supplement: Supplementary file 1 [file Data_Sheet_1.zip › Figure7/S-ARFPN-ResNet101/layer3 pyramid.png]

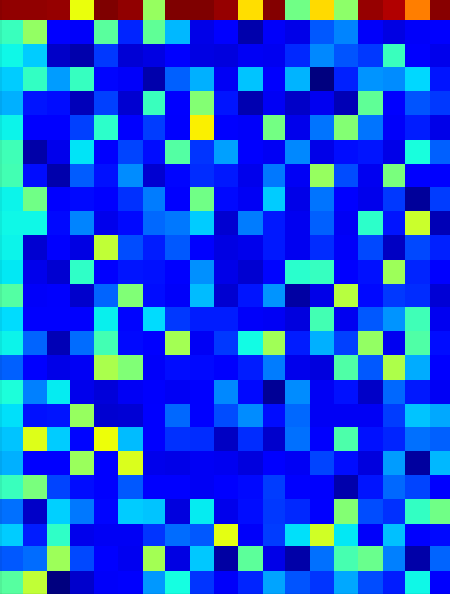

Supplement: Supplementary file 1 [file Data_Sheet_1.zip › Figure7/S-ARFPN-ResNet101/layer4 conv.png]

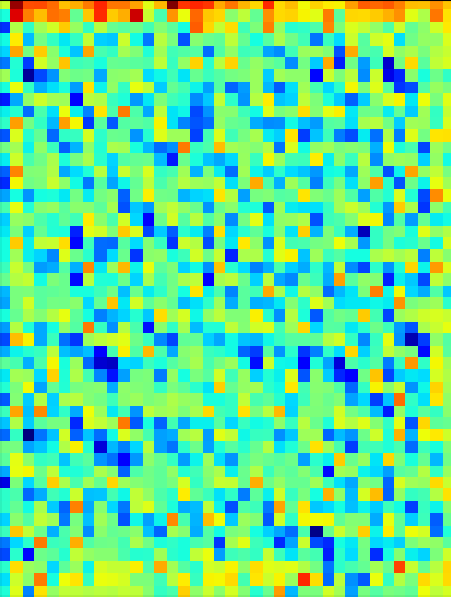

Supplement: Supplementary file 1 [file Data_Sheet_1.zip › Figure7/S-ARFPN-ResNet101/layer4 pyramid.png]

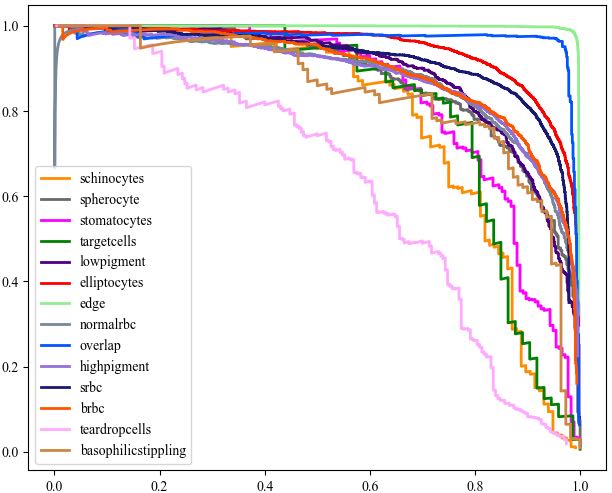

Supplement: Supplementary file 1 [file Data_Sheet_1.zip › Figure8/CS-ARFPN-ResNet50.jpg]

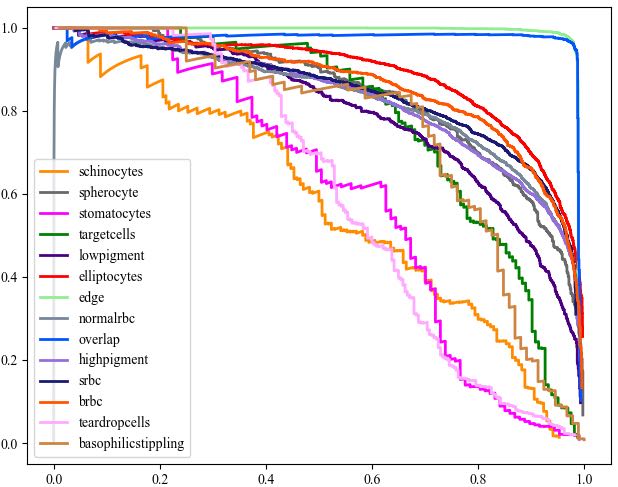

Supplement: Supplementary file 1 [file Data_Sheet_1.zip › Figure8/FPN-101.jpg]

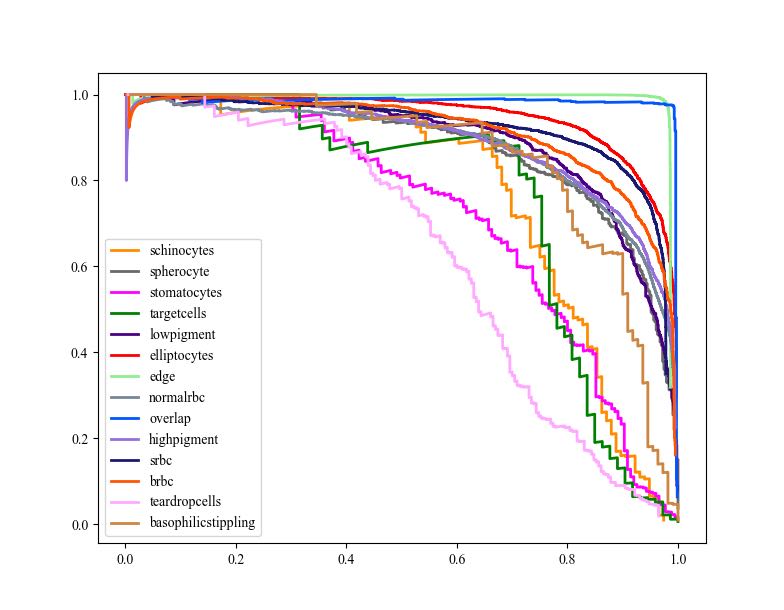

Supplement: Supplementary file 1 [file Data_Sheet_1.zip › Figure8/S-ARFPN-ResNet101.jpg]

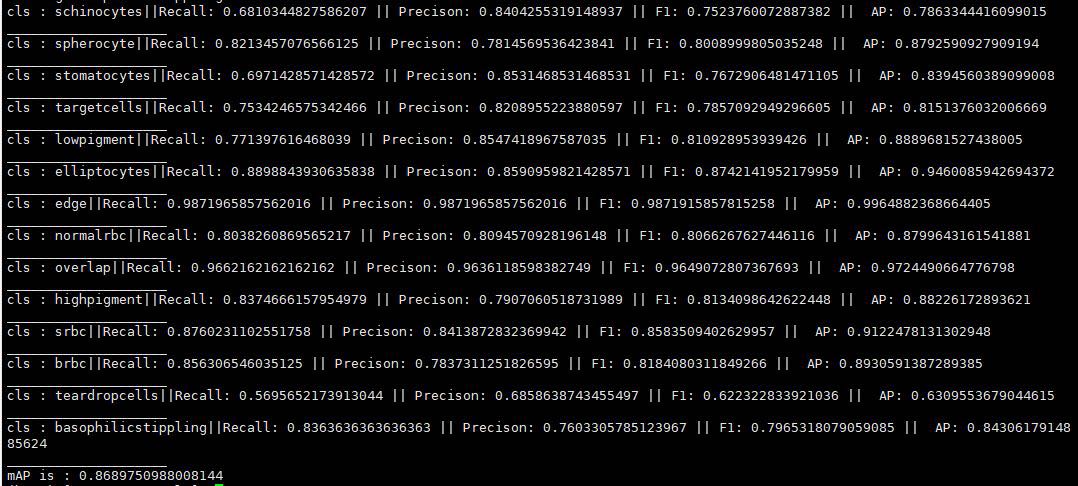

Supplement: Supplementary file 1 [file Data_Sheet_1.zip › Table3/CS-ARFPN-ResNet50.jpg]

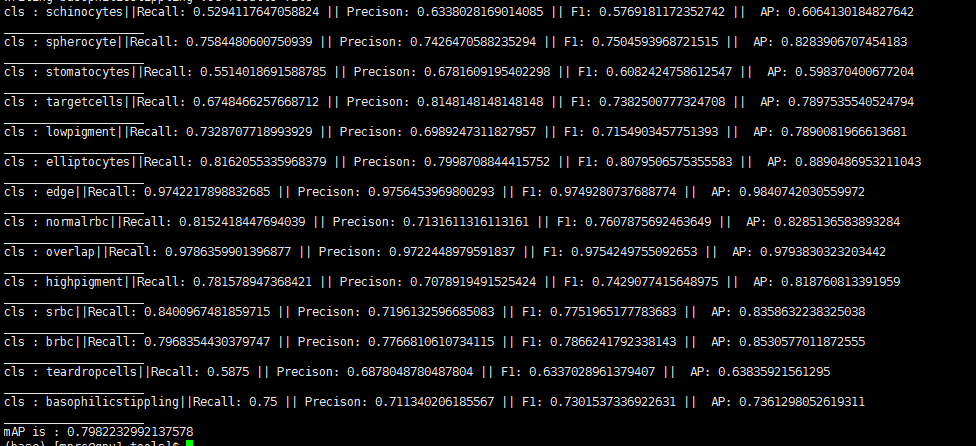

Supplement: Supplementary file 1 [file Data_Sheet_1.zip › Table3/FPN-ResNet101.png]

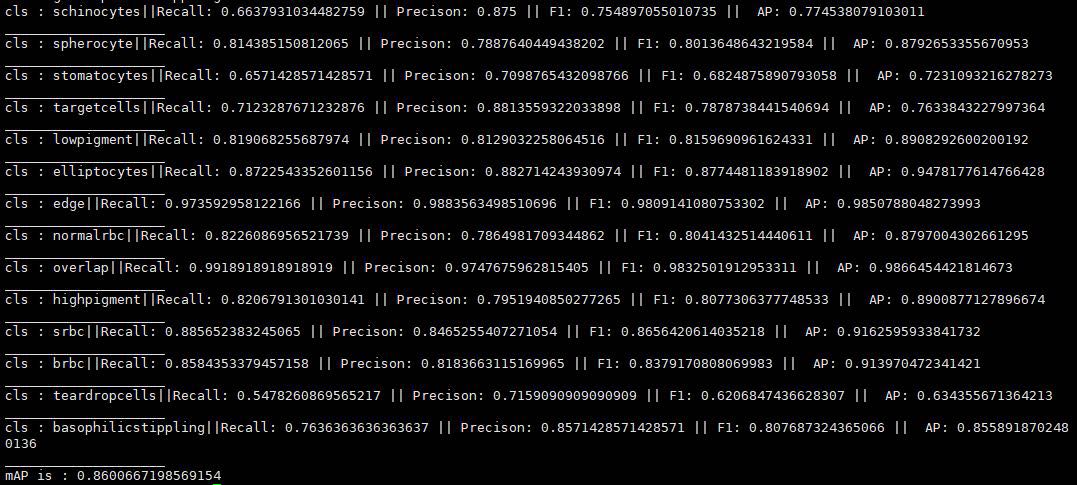

Supplement: Supplementary file 1 [file Data_Sheet_1.zip › Table3/S-ARFPN-ResNet101.jpg]

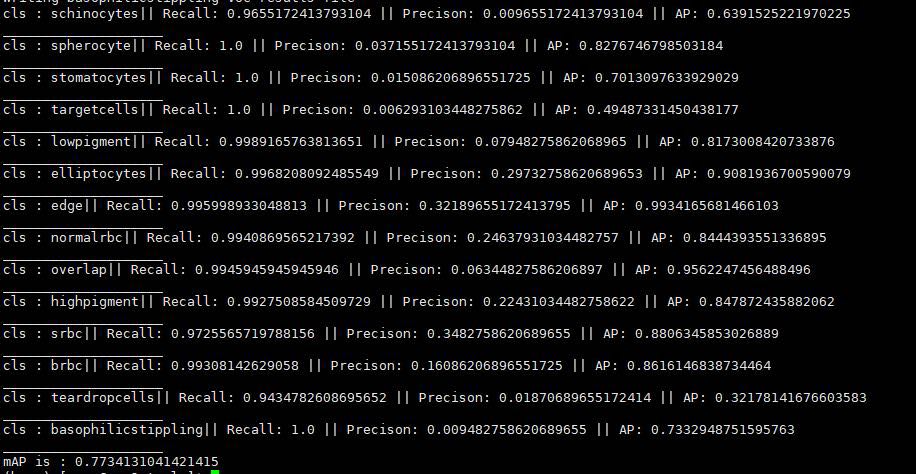

Supplement: Supplementary file 1 [file Data_Sheet_1.zip › Table5/Adam/CS-ARFPN-ResNet50.jpg]

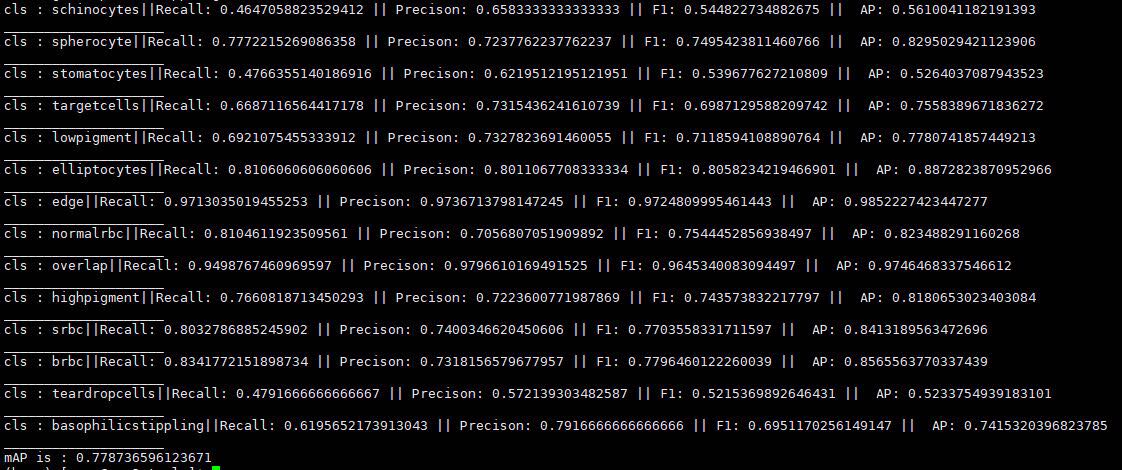

Supplement: Supplementary file 1 [file Data_Sheet_1.zip › Table5/Adam/FPN-ResNet101.jpg]

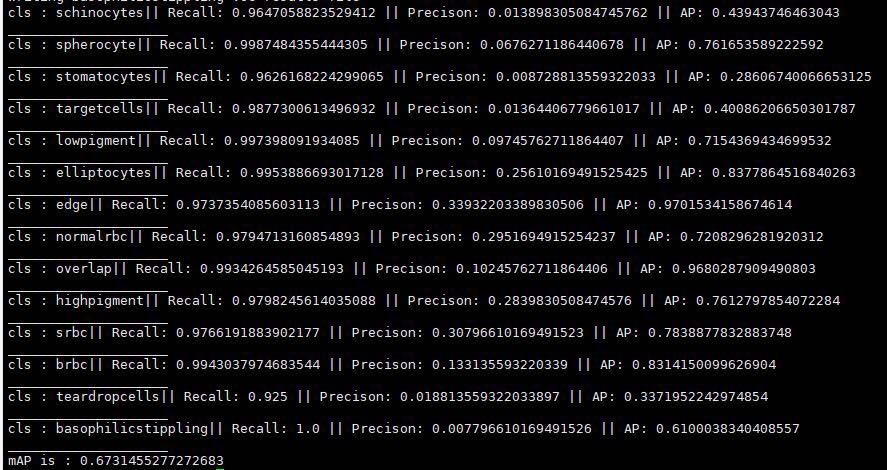

Supplement: Supplementary file 1 [file Data_Sheet_1.zip › Table5/Adam/S-ARFPN-ResNet101.jpg]

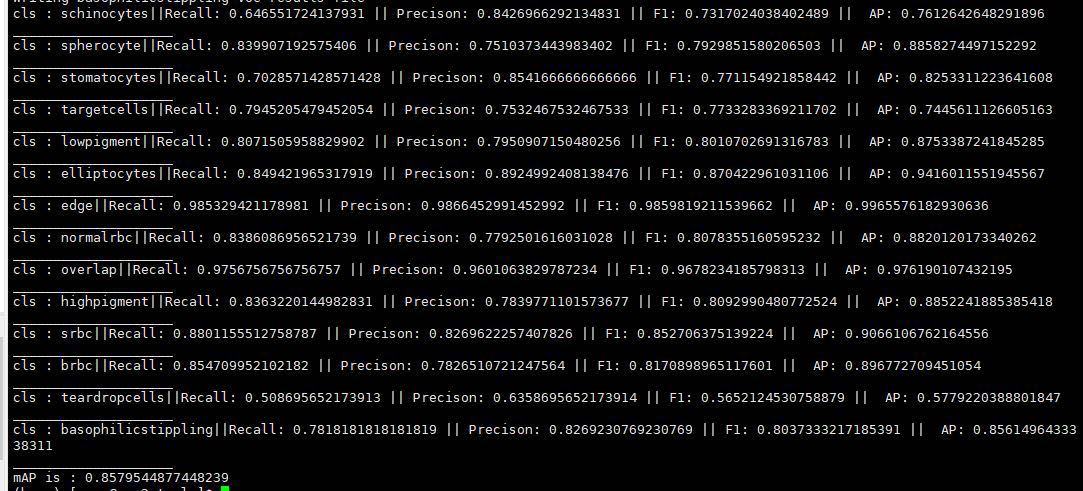

Supplement: Supplementary file 1 [file Data_Sheet_1.zip › Table6/RoI Pooling/CS-ARFPN-ResNet50.jpg]

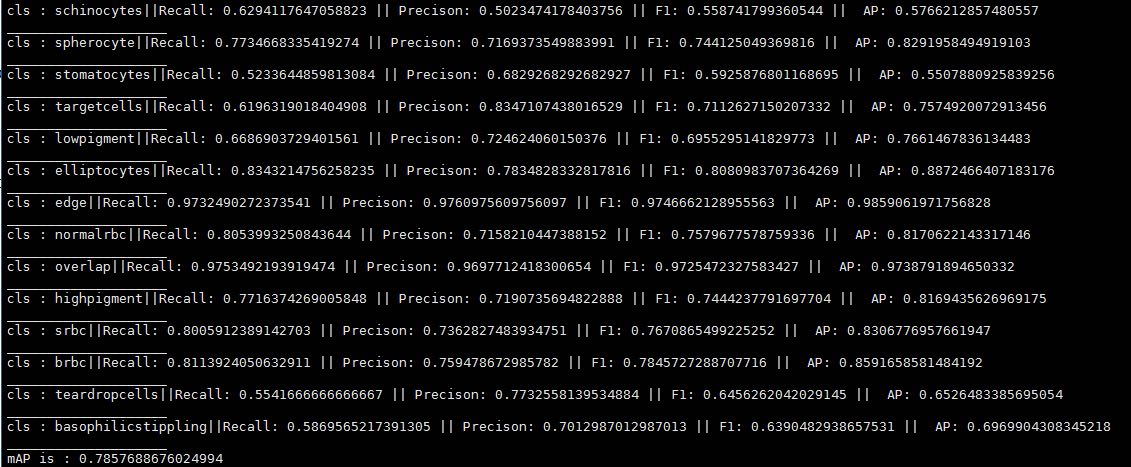

Supplement: Supplementary file 1 [file Data_Sheet_1.zip › Table6/RoI Pooling/FPN-ResNet101.jpg]

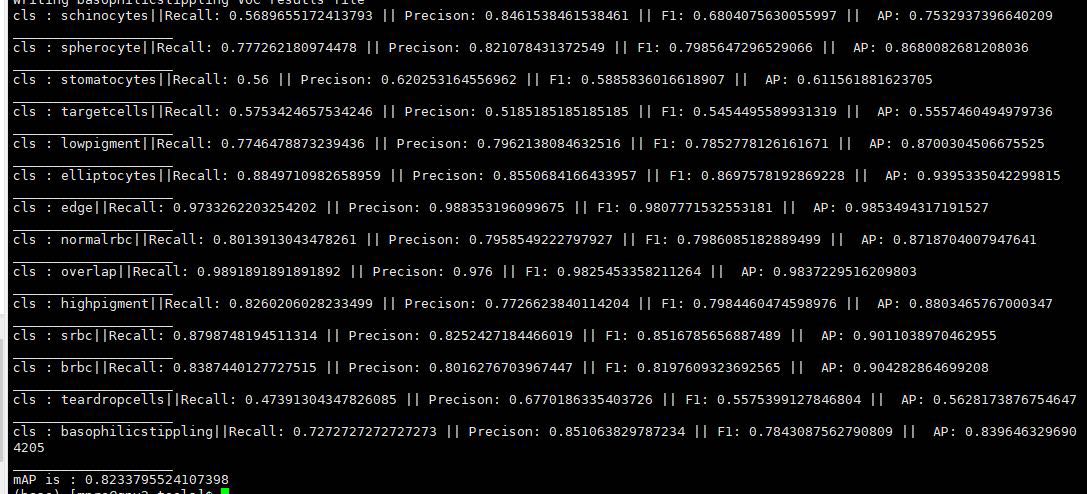

Supplement: Supplementary file 1 [file Data_Sheet_1.zip › Table6/RoI Pooling/S-ARFPN-ResNet101.jpg]

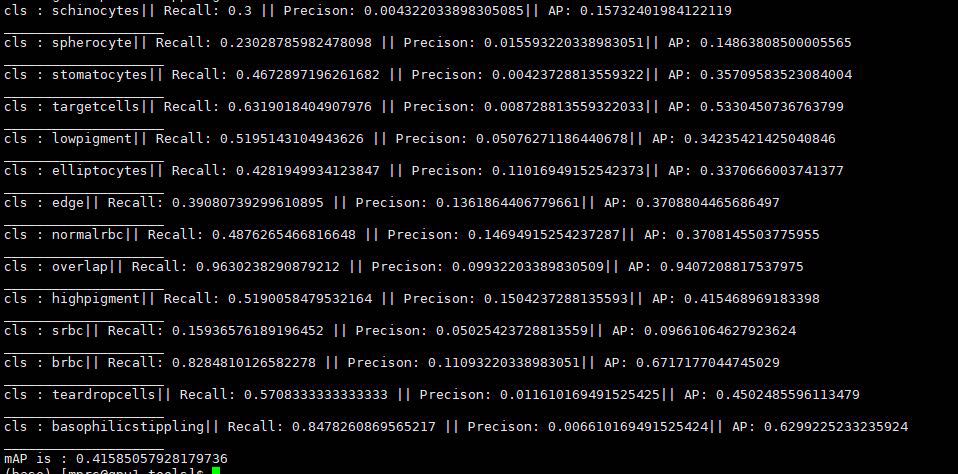

Supplement: Supplementary file 1 [file Data_Sheet_1.zip › Table7/ResNet-101/Cascade RCNN.jpg]

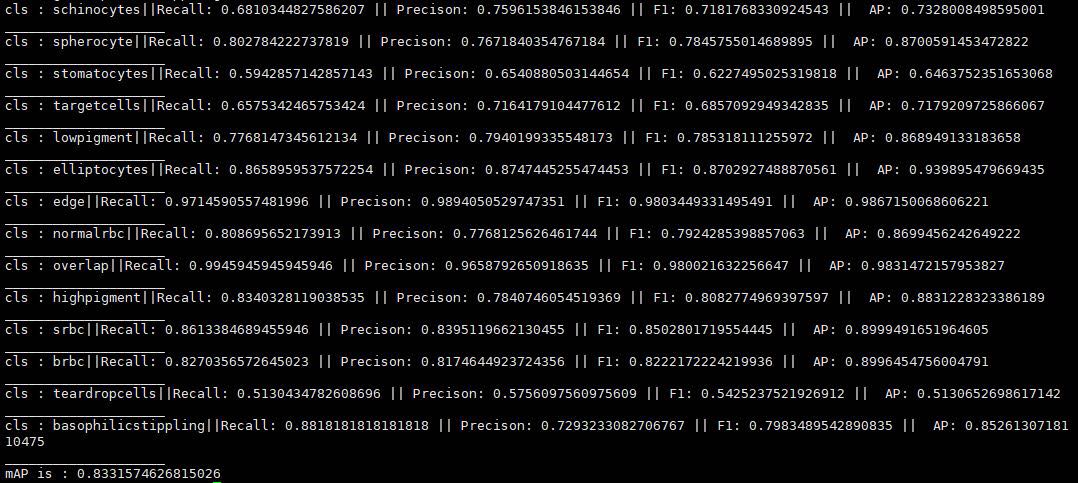

Supplement: Supplementary file 1 [file Data_Sheet_1.zip › Table7/ResNet-101/CS-ARFPN.jpg]

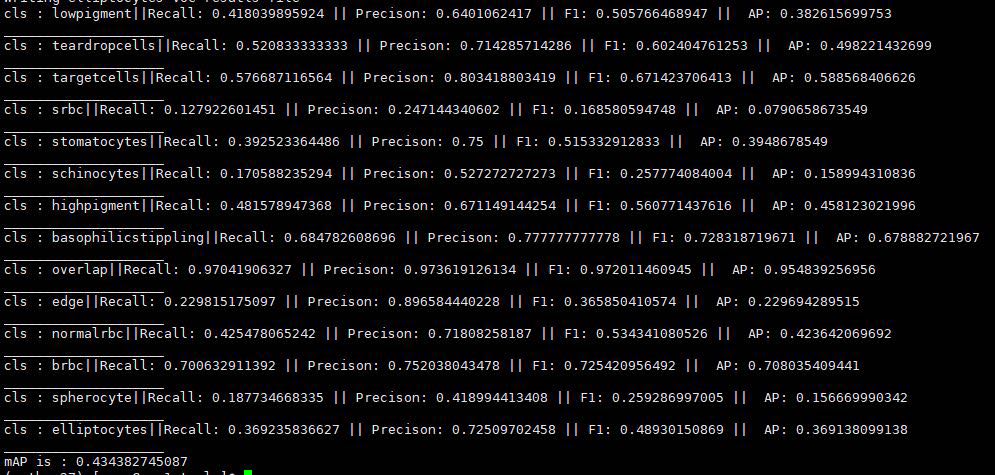

Supplement: Supplementary file 1 [file Data_Sheet_1.zip › Table7/ResNet-101/Faster RCNN.jpg]

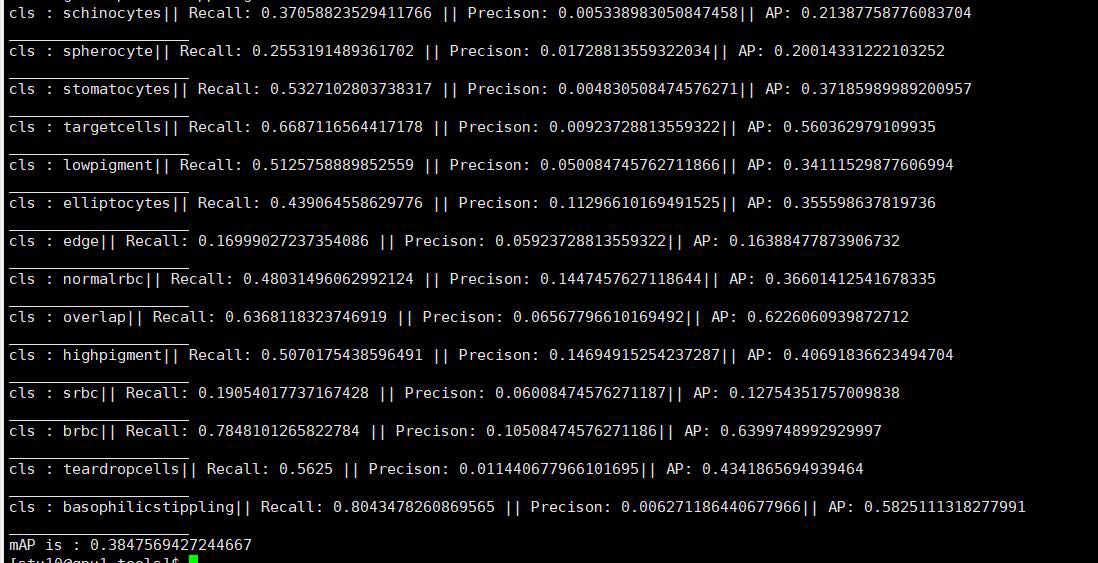

Supplement: Supplementary file 1 [file Data_Sheet_1.zip › Table7/ResNet-50/Cascade RCNN.jpg]

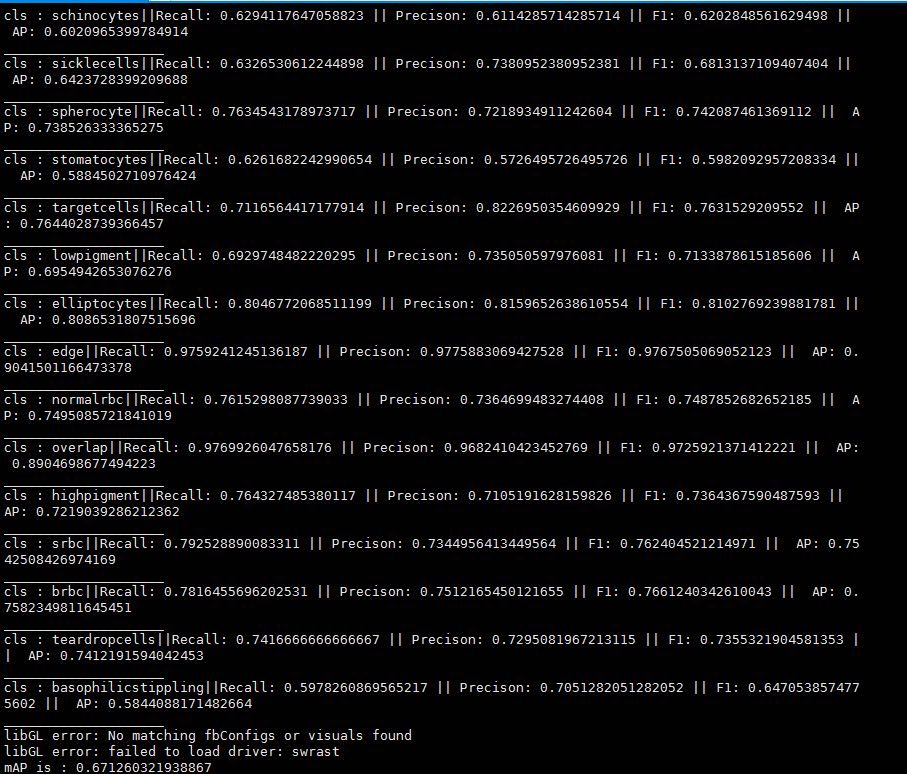

Supplement: Supplementary file 1 [file Data_Sheet_1.zip › Table7/ResNet-50/Cascade_FPN.jpg]

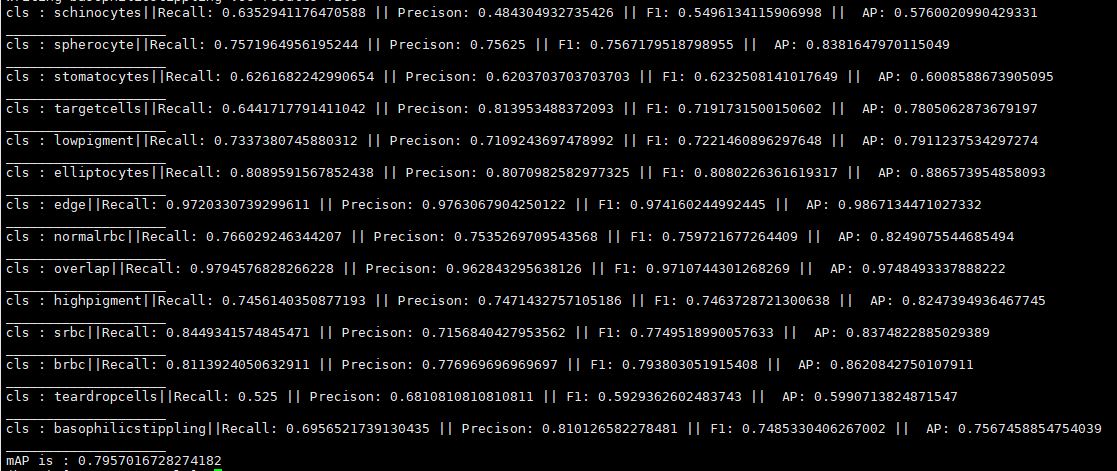

Supplement: Supplementary file 1 [file Data_Sheet_1.zip › Table7/ResNet-50/FPN.jpg]

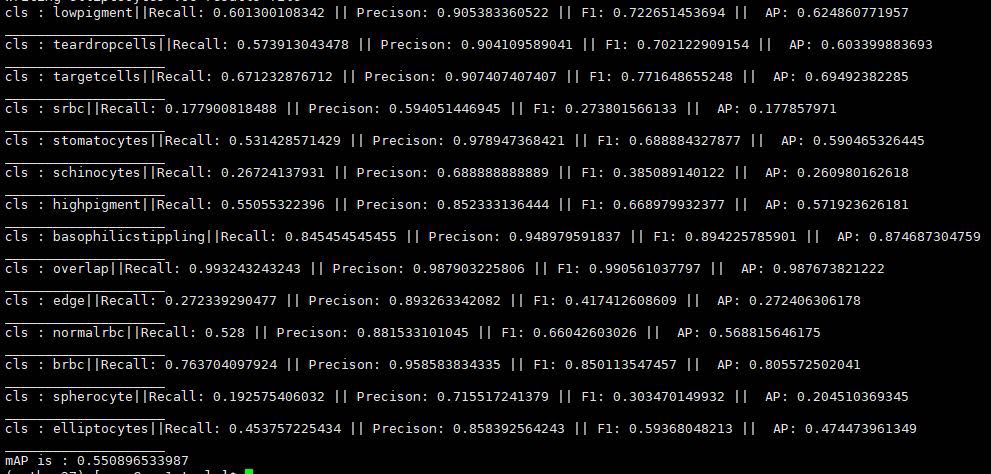

Supplement: Supplementary file 1 [file Data_Sheet_1.zip › Table7/ResNet-50/R-FCN.jpg]

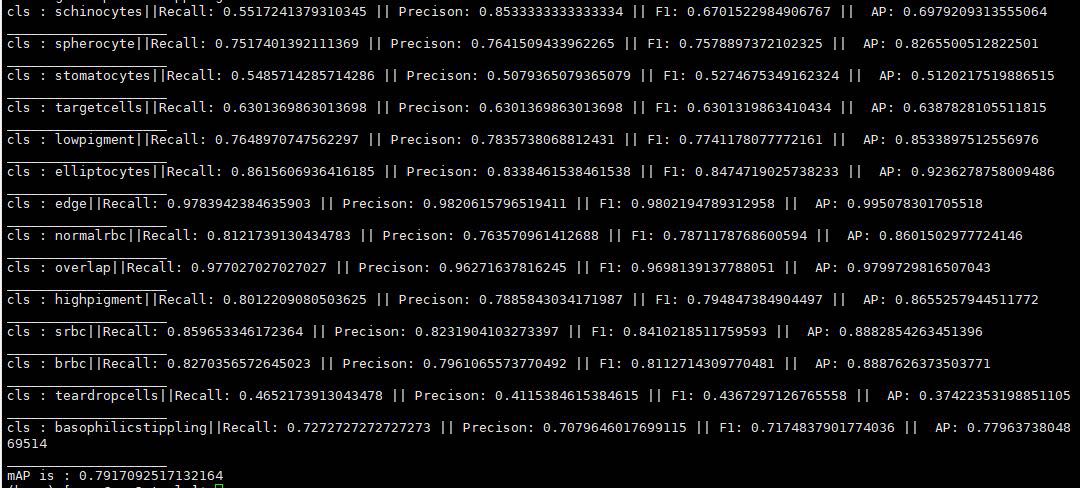

Supplement: Supplementary file 1 [file Data_Sheet_1.zip › Table7/ResNet-50/S-ARFPN.jpg]
